# Rapeseed (*Brassica napus*) Mitogen-Activated Protein Kinase 1 Enhances Shading Tolerance by Regulating the Photosynthesis Capability of Photosystem II

**DOI:** 10.3389/fpls.2022.902989

**Published:** 2022-06-02

**Authors:** Zhen Wang, Miao Liu, Mengnan Yao, Xiaoli Zhang, Cunmin Qu, Hai Du, Kun Lu, Jiana Li, Lijuan Wei, Ying Liang

**Affiliations:** ^1^Engineering Research Center for Rapeseed, College of Agronomy and Biotechnology, Southwest University, Chongqing, China; ^2^Engineering Research Center of South Upland Agriculture of Ministry of Education, Academy of Agricultural Sciences, Chongqing, China; ^3^Key Laboratory of Plant Resource Conservation and Germplasm Innovation in Mountainous Region (Ministry of Education), Institute of Agro-Bioengineering College, Guizhou University, Guiyang, China; ^4^Jiangsu Yanjiang Institute of Agricultural Sciences, Nantong, China

**Keywords:** *BnaMAPK1*, shading stress, photosynthesis, photosystem II, *Brassica napus*

## Abstract

Rapeseed (*Brassica napus*) is the third-largest source of vegetable oil in the world with an edible, medicinal, and ornamental value. However, insufficient light or high planting density directly affects its growth, development, yield, and quality. Mitogen-activated protein kinases (MAPKs) are serine/threonine protein kinases that play key roles in regulating the responses to biotic and abiotic stresses in plants. In this study, we found that the promoter of *BnaMAPK1* contained several light-responsive elements (including the AT1-motif, G-Box, and TCT-motif), consistent with its shading stress-induced upregulation. Compared with the wild type under shading stress, *BnaMAPK1*-overexpressing plants showed higher light capture efficiency and carbon assimilation capacity, enhancing their shading tolerance. Using RNA sequencing, we systematically investigated the function of *BnaMAPK1* in shading stress on photosynthetic structure, Calvin cycle, and light-driven electron transport. Notably, numerous genes encoding light-harvesting chlorophyll a/b-binding proteins (BnaLHCBs) in photosystem II-light-harvesting complex (LHC) II supercomplex were significantly downregulated in the *BnaMAPK1*-overexpressing lines relative to the wild type under shading stress. Combining RNA sequencing and yeast library screening, a candidate interaction partner of BnaMAPK1 regulating in shading stress, BnaLHCB3, was obtained. Moreover, yeast two-hybrid and split-luciferase complementation assays confirmed the physical interaction relationship between BnaLHCB3 and BnaMAPK1, suggesting that BnaMAPK1 may involve in stabilizing the photosystem II–LHC II supercomplex. Taken together, our results demonstrate that *BnaMAPK1* positively regulates photosynthesis capability to respond to shading stress in rapeseed, possibly by controlling antenna proteins complex in photosystem II, and could provide valuable information for further breeding for rapeseed stress tolerance.

## Introduction

Rapeseed (*Brassica napus*) is grown across the globe in various climatic conditions, from boreal to subtropical climates. It is utilized as leafy vegetable for human consumption, and also used for flower ornamental plant, oil production, biofuel, and animal feed (Confortin et al., 2019). The production of rapeseed is highly influenced by biotic and abiotic stresses, such as *Sclerotinia sclerotiorum*, *Peronospora parasitica*, light, temperature, drought, and salinity, all of which can drastically decrease yields (Raza et al., 2020; He et al., 2021; Raza, 2021); therefore, identifying stress-tolerance genes and understanding the related genetic network will provide a theoretical basis for developing new cultivars in rapeseed.

Plants can adapt to the complex environments by regulating gene expression and signal transduction pathways to advantageously alter their physiology and development. Mitogen-activated protein kinase (MAPK) cascades are highly conserved signal transduction pathways present in all eukaryotes (Cristina et al., 2010). A canonical MAPK network is composed of MAPK kinase kinases (MAPKKKs), MAPK kinases (MKKs), and MAPKs (Ichimura et al., 2002). The phosphorylated MAPKs in turn phosphorylate various downstream substrates, which are involved in the regulation of a wide variety of stress responses (Ichimura et al., 2002; Jonak et al., 2002; Pitzschke et al., 2009; Cristina et al., 2010; Zhang et al., 2018). MAPKs can be classified into four subfamilies (A–D), with subfamily C containing four members (MAPK1, MAPK2, MAPK7, and MAPK14) (Ichimura et al., 2002), which mainly play a role in abiotic stress responses. *AtMAPK1*, *AtMAPK2*, and *PsMAPK2* in pea (*Pisum sativum*) are all transcriptionally activated in response to wounding, jasmonic acid, and hydrogen peroxide (Ortiz-Masia et al., 2007, 2008). The four subfamily C MAPKs in *Arabidopsis* are activated by AtMKK3 to regulate the ABA signaling pathway (Danquah et al., 2015). It is likely that the MAPKs play varying roles in different stress responses (Teige et al., 2004).

Among several stress factors, light plays a vital role as it provides energy for photosynthesis and determines the growth, development, and morphogenesis of plants (Ruberti et al., 2012; Zoratti et al., 2014; Vialet-Chabrand et al., 2017). Insufficient light or shade condition is a pervasive abiotic stress in plant breeding and cultivation due to light blockage from intercropping, high planting density, horticulture facilities, cloud, rain, and snow. In rapeseed, shading stress is not conducive to photosynthetic efficiency, resulting in a decreased accumulation of dry matter and yield losses (Liang and Li, 2004). Photosynthate produced in the leaves, stem, and siliques are the primary nutrient source for rapeseed growth (Li et al., 2013; Lu et al., 2017), with the leaves being the most important until the flowering stage, when nutrients from the stem are also used for reproductive growth in the inflorescence (Li et al., 2013; Wang et al., 2016; Lu et al., 2017). At maturity, photosynthesis in the siliques facilitates the biosynthesis of proteins and lipids to store in the seeds (Elahi et al., 2016; Wang et al., 2016; Zafar et al., 2019). The reduction of photosynthesis under shading stress is therefore a major problem for rapeseed leaf and flower development and biomass accumulation, and overcoming this issue would be of great importance for breeding rapeseed with high edible, medicinal, and ornamental value.

In order to adapt to a shady environment, plants have evolved many strategies to increase their photosynthetic rate, including enhancing the stability of the photosystem I (PS I) and PS II complexes, the transcription, translation, and post-translational modification of photosynthesis-related genes and proteins (Walters, 2005). In higher plants, the light-harvesting complexes (LHCs) are divided into LHC I and LHC II groups, serving as PS I and PS II antennae, respectively (Klimmek et al., 2005, 2006). LHC II proteins are divided into four types (LHC II a–d), of which LHC II b is the major type. The three LHC II b proteins are encoded by the highly similar genes *LHCB1*, *LHCB2*, and *LHCB3*, and probably form homo- or hetero-trimers (Crepin and Caffarri, 2018). The minor LHC II proteins associated with PS II, CP29 (LHC II a), CP26 (LHC II c), and CP24 (LHC II d), are encoded by *LHCB4*, *LHCB5*, and *LHCB6*, respectively (Caffarri et al., 2009; Kirilovsky and Büchel, 2019). In the PS II complex, the inner antennae CP43 (PsbC) and CP47 (PsbB) bind to the D1 (PsbA) and D2 (PsbD) subunits to form core polypeptides, which then associate to the LHC II complex to form the PS II–LHC II supercomplex, playing a crucial role in capturing light in photosynthesis (Standfuss and Kühlbrandt, 2004).

The shading response affects the dynamic balancing of light transmission and distribution between PS I and PS II, which modulates the stability of the LHC II by the phosphorylation/dephosphorylation of protein kinases and phosphatases, increasing the light-harvesting area, and enhancing the efficiency with which light energy is used (Tikkanen and Aro, 2012; Rantala et al., 2020). Reversible and differential phosphorylation are dependent on the serine/threonine protein kinases 7 (STN7), STN8, and the thylakoid-associated phosphatase of 38-kD/protein phosphatase 1 (TAP38/PPH1) playing important roles in the phosphorylation of LHC II (Pribil et al., 2010; Tikkanen and Aro, 2012; Rantala et al., 2016, 2020). In addition, Zhang’s lab discovers that another type of serine/threonine protein kinases, AtMAPK3 and AtMAPK6, can rapidly downregulate various components in the PS II–LHC II supercomplex to participate in the hypersensitive response cell death in *Arabidopsis* (Su et al., 2018). In rapeseed, however, the effects of limited light and the molecular mechanisms of the shade response, especially the cross-link between MAPKs and LHCBs, remain undercharacterized and poorly understood.

Here, we isolated the promoter of *BnaMAPK1* (ProBnaMAPK1) and identified a series of light-responsive *cis*-acting elements, and tested the effect of shading on *BnaMAPK1* expression. Using wild-type (WT) and transgenic *BnaMAPK1*-overexpressing (*BnaMAPK1-*OE) rapeseed, the mechanisms of the *BnaMAPK1* response to shading stress were, respectively, investigated on photosynthetic structure (pigment and enzyme system and photosynthesis-related complex), Calvin cycle, and light-driven electron transport through the examination of a combination of photosynthetic traits, RNA sequencing (RNA-Seq), and protein–protein interactions. This research aims to characterize the function of BnaMAPK1 under shady conditions, determine how it may regulate shading tolerance, and identify the main signaling pathways and downstream targets of this MAPK in rapeseed. This study may provide new insights into MAPK cascades and help us to better understand the underlying biological process in a limited-light environment.

## Materials and Methods

### Plant Materials and Growth Conditions

The black-seed doubled haploid inbred *B. napus* cultivar Zhongyou821 (WT group), three *BnaMAPK1*-OE T_3_ lines (OE group), and tobacco (*Nicotiana benthamiana*) used in this study were obtained from Southwest University in China. The complete coding sequence of *BnaMAPK1* (*BnaA06g06010D*) under the control the CaMV *35S* promoter was transformed into Zhongyou821 to create OE materials with Basta resistance, as described previously (Weng et al., 2014). The rapeseed and tobacco seeds were soaked in Petri dishes and stratified at 4°C for 2–3 days. The seeds were then sown in nutritious soil in a growth chamber (PGR15, Controlled Environments, Winnipeg, MB, Canada), with a 16-h light (25°C)/8-h dark (20°C) photoperiod, 75% humidity, and an 800 μmol·m^−2^·s^−1^ light intensity. The photosynthetic photon flux density (PPFD) was measured using a quantum radiometer/photometer (LI-185B; LI-COR Biosciences, Lincoln, NE, United States).

### Photosynthetic Light-Response Curve Analysis and Shading Treatment

For the photosynthetic light-response curve measurements, three healthy and mature leaves from the center of each 3–4-week-old Zhongyou821 seedling at the six-leaf stage (with four expand true leaves and two bud leaves) were selected and marked as fixed measured leaves. Curves were measured 10 times for each marked leaf, so that 30 times were measured for each seedling, with three seedlings used for each light intensity. During the measurement, the CO_2_ concentration was set to the atmospheric CO_2_ concentration, the flow rate of air in the measuring chamber was about 500 μmol·s^−1^, the temperature of the leaf chamber was 25°C ± 1°C, and the relative humidity was 75% ± 5%. For every measurement, the photosynthetically active radiation (PAR) was set at 1,400, 1,200, 1,000, 800, 500, 400, 300, 200, 100, 50, 20, and 0 μmol·m^−2^·s^−1^ by portable photosynthetic system (LI-6400; LI-COR Biosciences) to measure the net photosynthetic rate (Pn) under different light intensities (Li et al., 2019). For each PAR, the measurement time was controlled to 4 min and Pn was stabilized and recorded automatically by the instrument. The light saturation point (LSP), light compensation point (LCP), dark respiration rate (Rd), maximum net photosynthetic rate (Pnmax), and apparent quantum efficiency (AQE) were estimated based on the trend of the light-response curve by the modified rectangular hyperbola model (Ye et al., 2013).

Based on the photosynthetic light-response curve, a light intensity of 300 μmol·m^−2^·s^−1^ was selected to perform the shading treatment on the WT and three independent *BnaMAPK1-*OE lines (OE-1, OE-2, and OE-3) at the six-leaf stage. These plants were exposed to the shading treatment for 0, 7, 14, 21, and 28 days to investigate their photosynthetic traits under this stress. For the analysis of *BnaMAPK1* expression, the leaves of the WT seedlings were, respectively, collected after 0, 1.5, 3, 6, 9, 12, and 15 h of the shading treatment and used for RNA extraction. Leaves were also collected after 0 and 12 h of treatment for RNA-Seq. These leaves samples were transferred into RNase-free microfuge tubes and immediately placed into liquid nitrogen. Three biological duplicates were performed for each experiment.

### Photosynthetic Trait Measurements

For the measurements of the physiological and chlorophyll fluorescence traits, WT and *BnaMAPK1-*OE leaves after 0 and 28 days of shading stress were measured using a LI-6400 portable photosynthetic system, with the same settings as were used for the light-response curve (Zhao et al., 2015; Pan et al., 2018). Each leaf was tested 10 times, and the average values after 0 and 28 days of the shading treatment were calculated. The biochemical traits include the Pn, stomatal conductance (Gs), and intercellular CO_2_ concentration (Ci), and transpiration rate (Tr). For the chlorophyll fluorescence measurements, all plants were dark-adapted in advance for at least 3 h. The minimal fluorescence (F0), photochemical quenching coefficient (qP), non-photochemical quenching coefficient (qN), and maximum photochemical efficiency of PS II (Fv/Fm) were measured in each leaf and the average values were calculated (Li et al., 2020).

For the measurements of the biochemical traits, the relative content of chlorophyll (SPAD value) was determined in the WT and *BnaMAPK1-*OE by means of a chlorophyll meter (SPAD-502; Konica Minolta, Tokyo, Japan). Each leaf was marked at 10 representative points, and the average SPAD values after 0, 7, 14, 21, and 28 days of the shading treatment were calculated (Li et al., 2020). The Ribulose-1,5-bisphosphate carboxylase/oxygenase (Rubisco) activity was assayed as described in the protocol of the plant Rubisco enzyme-linked immunosorbent assay kit (Cusabio Biotech, Wuhan, China). Fresh leaf material (0.5 g) was obtained from the WT and *BnaMAPK1-*OE seedlings after 0, 7, 14, 21, and 28 days of the shading treatment. Using the slightly modified enzyme extraction method of Xu et al., (2020), the material was homogenized with 6 ml of cooled extraction buffer containing 100 mM Tris–HCl (pH 7.8), 10 mM MgCl_2_, 1 mM EDTA, 20 mM β-hydroxy-1-ethanethiol, and 2% (m/v) polyvinyl pyrrolidone at 0°C–4°C. The product was centrifuged at 14,000× *g* for 20 min at 4°C, and the supernatant was used for Rubisco activity measurement.

### RNA Isolation, cDNA Biosynthesis, and Real-Time Quantitative PCR Analyses

To identify the relative expression levels of *BnaMAPK1* during shading stress, Zhongyou821 seedling leaf samples were harvested after 0, 1.5, 3, 6, 9, 12, and 15 h of the shading treatment. Total RNA was extracted from non-stressed and shade-stressed rapeseed seedling leaves using the RNAprep pure plant kit (Tiangen Biochemical Technology, Beijing, China), and reverse transcription reaction was performed by the PrimeScript RT reagent kit (Takara Bio, Tokyo, Japan). The real-time quantitative PCR (qRT-PCR) was performed on a qTOWER2.2 qRT-PCR thermal cycler system (Analytik Jena, Jena, Germany). All qRT-PCRs were conducted in triplicate biological replications, and the data were comparatively quantified and calibrated using the qPCRsoft 3.1 system (Analytik Jena). The expression level of each gene was determined by the 2^−ΔΔCt^ method (Livak and Schmittgen, 2001), using rapeseed *BnaACT7* (*BnaC02g00690D*) expression as the internal control (Liu et al., 2010). At least three independent biological repeats were performed for each data set. All the primers used to detect the gene expression are listed in Supplementary Table S1.

### RNA-Seq Analysis

The RNA-Seq analysis was performed on WT and *BnaMAPK1*-OE rapeseed plants after 0 and 12 h of shading stress. The leaves, stems, and roots of seedlings were collected as mixed samples for total RNA extraction. The RNAs of three OE lines were extracted, respectively, and mixed with equal quality by three biological replicates, named as OE-Rep1, OE-Rep2, and OE-Rep3. Libraries were constructed and sequenced at Beijing Biomarker Technology (Beijing, China). An Illumina Hiseq X 10 sequencing platform was used to generate paired-end reads. Sequencing data has been deposited in the Sequence Read Archive (SRA) of the National Center for Biotechnology Information (NCBI) database with the accession BioProject ID: PRJNA680826.

The raw data were first processed to perform quality control to obtain clean reads which were then mapped to the *B. napus* reference genome sequence with Hisat2 tools (Kim et al., 2015). The gene expression levels were estimated using the fragments per kilobase of transcript per million fragments mapped (FPKM) method (Mortazavi et al., 2008). A differential expression analysis of the control/shading-treated plants and the *BnaMAPK1*-OE/WT groups was performed using DESeq (Wang et al., 2009). The following pairwise comparisons were performed: *BnaMAPK1*-OE samples with WT samples under the control (0h_OE vs. WT) and shading treatments (12h_OE vs. WT), and WT plants (WT_12h vs. 0 h) or *BnaMAPK1*-OE plants (OE_12h vs. 0 h) subjected to the control and stress conditions. Differentially expressed genes (DEGs) were defined as having a FPKM value > 1 for each of the three biological replicates, an absolute fold change (FC) value ≥ 2, and false discovery rate (FDR) < 0.05. The enrichment analysis of Gene Ontology (GO) and Kyoto encyclopedia of genes and genomes (KEGG) pathways of the DEGs in the 12h_OE vs. WT group was implemented by GOseq R packages with a *p.adjust* < 0.05 (Young et al., 2010) and KOBAS software with a *q-value* < 0.05 (Mao et al., 2005), respectively. qRT-PCR was performed to verify the RNA-Seq data (primers are listed in Supplementary Table S1).

### Yeast Two-Hybrid and Split-Luciferase Complementation Assays

For the Yeast two-hybrid (Y2H) assays, the open reading frames of *BnaLHCB3* (*BnaA10g07350D*) and *BnaMAPK1* were amplified from Zhongyou821 and ligated into the pGADT7 prey vector and pGBKT7 bait vector, respectively. The confirmed *BnaLHCB3* and *BnaMAPK1* constructs were cotransformed into Y2Hgold cells and then plated onto SD-Leu-Trp medium and SD–Ade-His-Leu-Trp medium to observe the protein interactions. The interaction between pGBKT7-53 and pGADT7-T was the positive control, while the pGBKT7-Lam and pGADT7-T pair was used as the negative control.

For the split-Luciferase complementation (split-LUC) assays, *BnaLHCB3* and *BnaMAPK1* were recombined into the pCAMBIA1300-nLUC and pCAMBIA1300-cLUC vectors, respectively, which were then independently transformed into *Agrobacterium tumefaciens* strain GV3101. Cells were suspended in infiltration buffer (10 mM MgCl_2_, 100 μM acetosyringone, 10 mM MES; pH 5.8) to an OD_600_ of 0.4–0.6 and incubated in the dark for at least 3 h at 28°C. Corresponding cLUC and nLUC cells were equally mixed and infiltrated into tobacco leaves. After 2 days, leaves were collected to examine LUC activity with 1 mM of the LUC substrate D-luciferin (APExBIO Technology, Houston, TX, United States). Luminescence intensity was measured by a CCD imaging apparatus (IVIS Lumina XRMS Series III; PerkinElmer, Waltham, MA, United States) with an exposure time of 2 min and binning settings 3 × 3. pCAMBIA1300-nLUC and pCAMBIA1300-cLUC were used as the negative and reference controls to normalize the LUC activity and eliminate variation in the experiment. The relative LUC activity values were obtained for three independent samples and used to calculate the arithmetic mean, as previously reported (Hou et al., 2016; Zhu et al., 2017). Primers are listed in Supplementary Table S1.

### Statistical Analysis

All experiments were assessed using at least three independent repeats, and all data are represented as the mean ± SD. The statistical analysis was performed using a Student’s *t*-test, and the significance was indicated using asterisks: ^*^(*p-value* < 0.05) or ^**^(*p-value* < 0.01).

## Results

### *BnaMAPK1* Can Be Induced by Shading Stress in Rapeseed

To identify the putative *cis*-acting regulatory elements in the promoter of *BnaMAPK1* in the Zhongyou821 rapeseed line, a 1,389-bp sequence upstream of the start codon was obtained using a PCR amplification and cloned based on the promoter of *BnaA06g06010D* from the Genoscope database. A bioinformatics analysis of the promoter sequence using the PlantCARE online tools (Lescot et al., 2002) revealed the presence of *cis*-acting elements that were shown to regulate gene expression in response to stress in plants. Several light-responsive elements were also identified, including the AT1-motif (ATTAATTTTACA), G-box (CACGTC), and TCT-motif (TCTTAC), suggesting that *BnaMAPK1* may be involved in responding to light stress. The detected putative light-responsive elements and their functions are listed in Supplementary Table S2.

Using a quantum radiometer/photometer, the Pn of Zhongyou821 rapeseed was measured to investigate the light-response curve. Before the PAR reached 800 μmol·m^−2^·s^−1^, the Pn increased markedly, although little change was observed when the PAR was between 300 and 400 μmol·m^−2^·s^−1^ (Supplementary Figure S1A). Using the Ye Zi-Piao model to fit the light-response curve, the fitted result was similar to the actual measured value, with 800 μmol·m^−2^·s^−1^ set as the light saturation point and 300 μmol·m^−2^·s^−1^ as the shading stress light intensity (Supplementary Table S3). To detect the response patterns of *BnaMAPK1* expression, the plants were subjected to 15 h of shading stress at 300 μmol·m^−2^·s^−1^. A qRT-PCR analysis showed that *BnaMAPK1* was upregulated after 3 h, reached a peak at 12 h, then decreased, indicating that *BnaMAPK1* was induced by shading stress (Supplementary Figure S1B).

### *BnaMAPK1* Responds to Shading Stress and Improves Shading Tolerance

**Figure 1:**
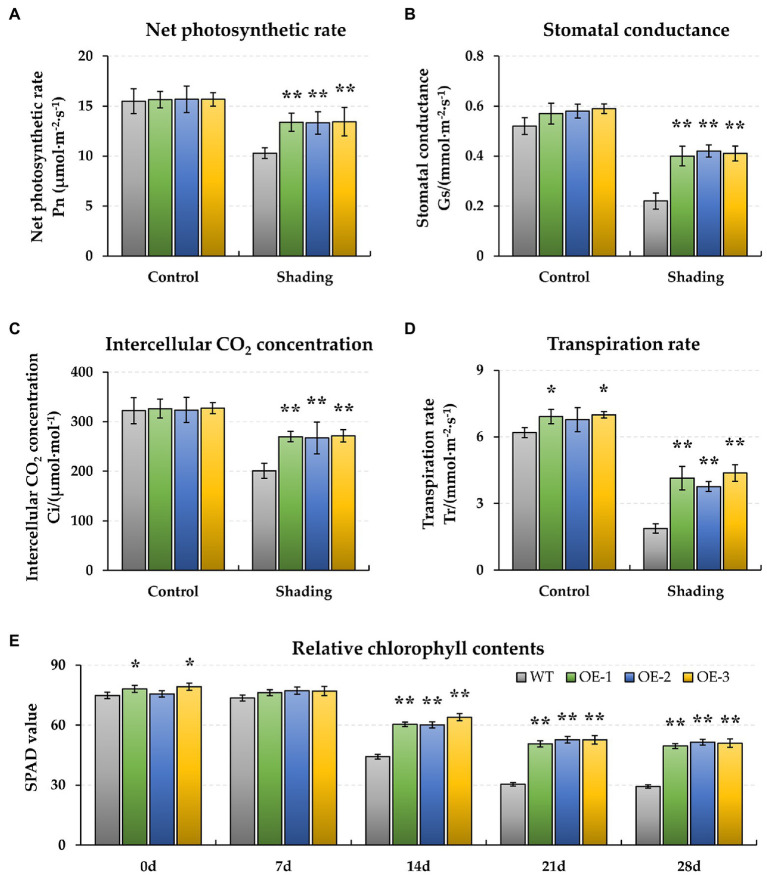
Effect of shading stress on the physiological photosynthesis and pigment of wild type (WT) and *BnaMAPK1*-overexpressing (*BnaMAPK1*-OE) transgenic rapeseed. **(A)** Net photosynthetic rate (Pn). **(B)** Stomatal conductance (Gs). **(C)** Intercellular CO_2_ concentration (Ci). **(D)** Transpiration rate (Tr). **(E)** Relative chlorophyll contents (SPAD). The physiological parameters (Pn, Gs, Ci, and Tr) were measured under normal light (control) and shading conditions at 0 and 28 days each, and the chlorophyll content (SPAD) at 0, 7, 14, 21, and 28 days each. Vertical bars are means of three replicates ± SD (three plants of each line, three leaves of each plant, and 10 times of each leaf were measured); asterisk and double asterisk indicate *p-value* < 0.05 and *p-value* < 0.01.

To evaluate the role of *BnaMAPK1* in protecting the photosynthetic apparatus from shading stress, we produced *BnaMAPK1*-OE lines of rapeseed. In the three overexpression independent lines (OE-1, OE-2, and OE-3), the relative expression levels of *BnaMAPK1* were significantly higher than in WT rapeseed (Supplementary Figure S1C). To investigate how the rapeseed plants were affected under shading stress, the physiological traits of WT and *BnaMAPK1-*OE rapeseed were determined. Under normal light conditions (Control, 0 days), the Pn, Gs, and Ci displayed no significant differences between *BnaMAPK1-*OE and WT rapeseed, although the Tr values of the *BnaMAPK1-*OE-1 and *BnaMAPK1-*OE-3 lines were significantly higher than that of the WT (*p-value* < 0.05). When the plants were exposed to the shading treatment for 28 days, the Pn of both the *BnaMAPK1-*OE and WT lines decreased markedly, although the decrease was greater in the WT than in the *BnaMAPK1-*OE plants (*p-value* < 0.01) (Figure 1A), suggesting that overexpressing *BnaMAPK1* could alleviate the decrease in photosynthetic rate caused by shading stress. In addition, the observed changes in Gs, Ci, and Tr were similar to Pn, with significantly greater decreases observed in the WT than the transgenic plants under shading stress (Figures 1B–D). These data indicated that overexpressing *BnaMAPK1* enhances the gas exchange ability of rapeseed to improve its photosynthetic rate under long-term low-light environments.

**Figure 2:**
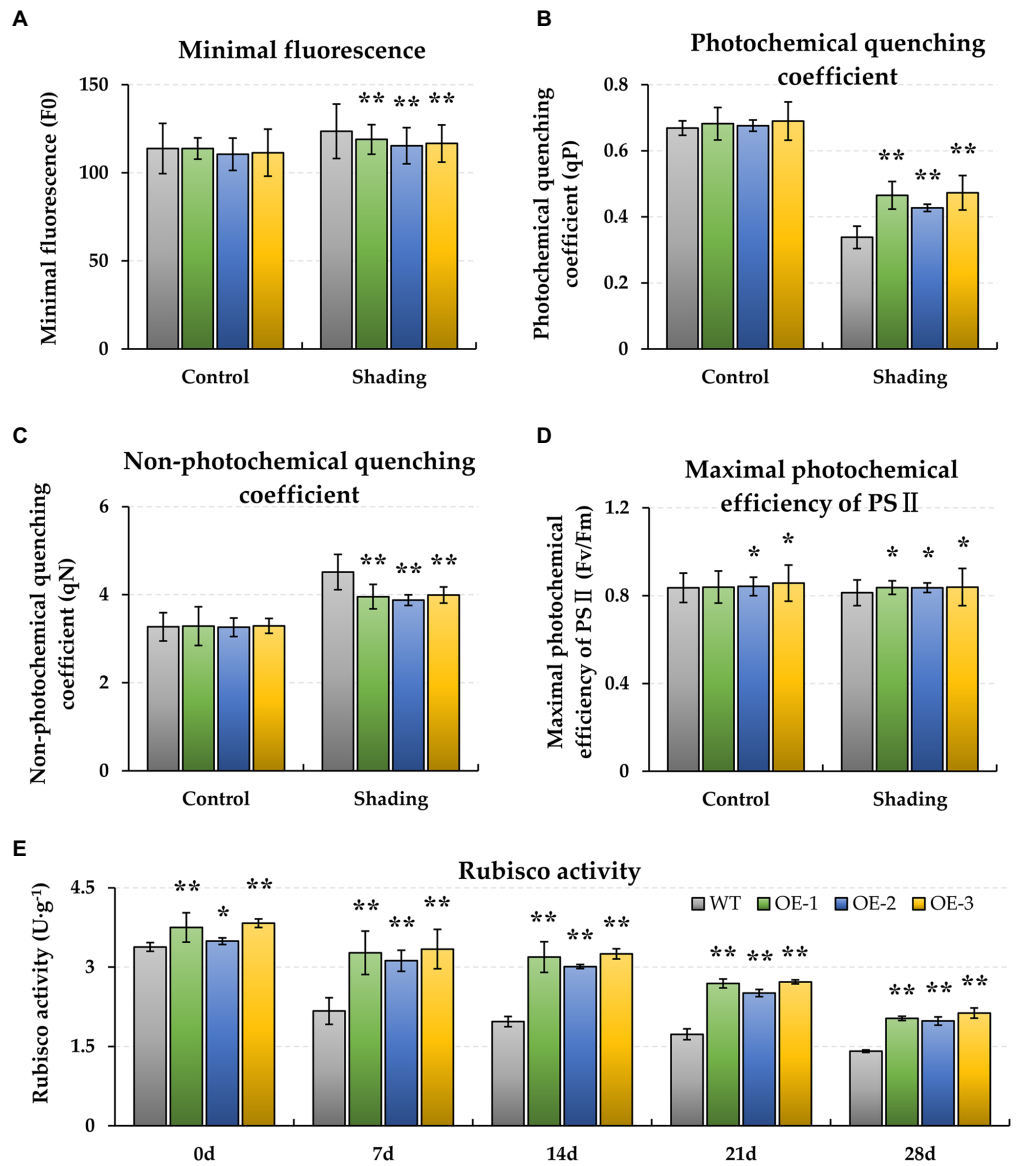
Effect of shading stress on the chlorophyll fluorescence photosynthetic traits and Rubisco activity of WT and *BnaMAPK1*-OE transgenic rapeseed. **(A)** Minimal fluorescence (F0). **(B)** Photochemical quenching coefficient (qP). **(C)** Non-photochemical quenching coefficient (qN). **(D)** Maximum photochemical efficiency of PS II (Fv/Fm). **(E)** Rubisco activity. The chlorophyll fluorescence parameters (F0, qP, qN, and Fv/Fm) were measured under control and shading conditions at 0 and 28 days each, and the Rubisco activity at 0, 7, 14, 21, and 28 days each. Vertical bars are means of three replicates ± SD (three plants of each line, three leaves of each plant, and 10 times of each leaf were measured); asterisk and double asterisk indicate *p-value* < 0.05 and *p-value* < 0.01.

To observe the energy dissipation, the chlorophyll fluorescence characteristics of F0, qP, qN, and Fv/Fm were measured. When plants were exposed to shading stress for 28 days, the F0 and qN increased in both the WT and *BnaMAPK1*-OE rapeseed relative to the normal light condition, with these traits remaining significantly lower in the *BnaMAPK1*-OE lines than in the WT under the shaded condition (*p-value* < 0.01) (Figures 2A,C). This suggests that overexpressing *BnaMAPK1* enhances the light capture efficiency to improve plants’ sensitivity to light. Additionally, after 28 days of shading, qP and Fv/Fm decreased in the WT and *BnaMAPK1*-OE plants, indicating a decrease in the rate of light energy conversion. Both qP and Fv/Fm remained higher in the *BnaMAPK1*-OE lines than in the WT plants under shading stress, demonstrating that the overexpression of *BnaMAPK1* could effectively maintain the transmission of light in PS II to regulate photosynthetic ability during shading stress (Figures 2B,D).

Additionally, the biochemical characteristics of WT and *BnaMAPK1*-OE rapeseed were also assessed under shading stress. As shown in Figure 1E, the relative chlorophyll contents of the *BnaMAPK1*-OE lines were all significantly higher (*p-value* < 0.01) than the WT after 14 days of shading stress (*BnaMAPK1*-OE plants: 61.52, 52.01, and 50.66; WT: 44.20, 30.35, and 29.33 after 14, 24, and 28 days of shading). This demonstrated that overexpressing *BnaMAPK1* led to the retention of more chlorophyll, increasing the light energy utilization under shading stress in rapeseed. Rubisco is a key enzyme for photosynthesis, and was therefore investigated next. The activities of Rubisco in the three *BnaMAPK1-*OE lines were significantly higher than in the WT plants under normal light condition, while the shading treatments decreased the Rubisco activity of both the *BnaMAPK1-*OE and WT lines, although the *BnaMAPK1-*OE lines remained markedly higher than that of the WT (Figure 2E). These findings suggest that overexpressing *BnaMAPK1* could improve Rubisco activity, which would affect the net photosynthetic rate. Our data therefore confirmed that *BnaMAPK1* positively regulates light capture efficiency and carbon assimilation capacity to improve shading tolerance in rapeseed, which was consistent with the results of the promoter *cis*-acting element analysis.

### Identification of DEGs Under Shading Stress Using RNA-Seq

**Figure 3:**
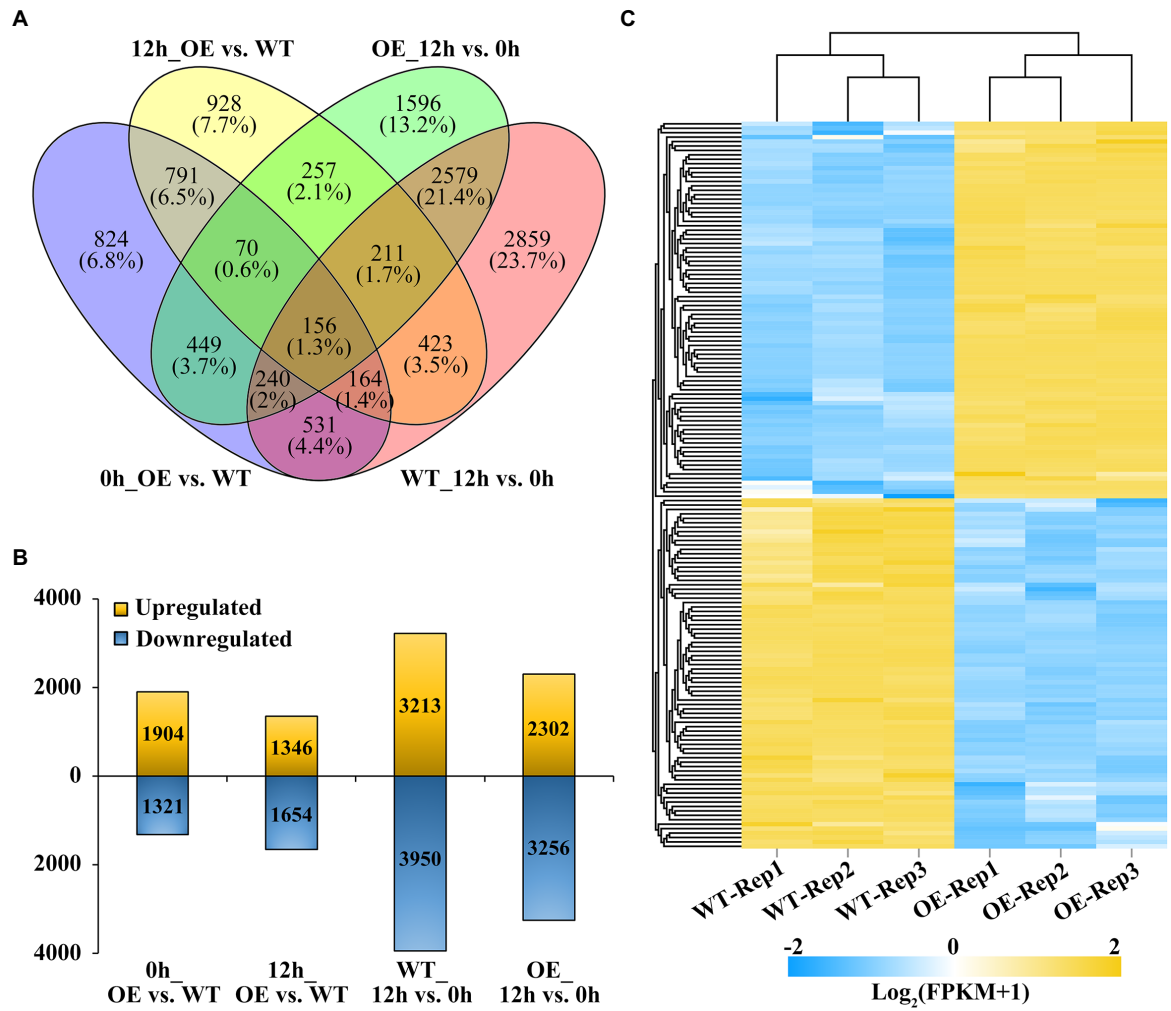
Comparison of significant differentially expression genes (DEGs) identified in *BnaMAPK1*-OE and WT rapeseed under shading stress. **(A)** Venn diagram of DEGs in total by four comparisons of WT with *BnaMAPK1*-OE samples at control and shading stress conditions. DEGs screened with FPKM value of three replicates > 1, |Log_2_FC| > 1, and FDR < 0.05 among each pairwise comparisons. **(B)** Statistics of the number of upregulated and downregulated DEGs in four comparisons of WT with *BnaMAPK1*-OE samples at control and shading stress. **(C)** Expression heatmap of significant DEGs in *BnaMAPK1-*OE and sWT rapeseed under shading stress. One hundred and sixty four DEGs screened with each FPKM value of three replicates > 1, |Log_2_FC| > 3, and FDR < 0.05. 0h_OE vs. WT, *BnaMAPK1*-OE samples with WT at normal light; 12h_OE vs. WT, *BnaMAPK1*-OE samples with WT at shading stress; WT_12h vs. 0 h, normal light samples with shading treatment samples in WT; OE_12h vs. 0 h, normal light samples with shading treatment samples in *BnaMAPK1*-OE lines.

To gain insight into the regulatory mechanisms by which *BnaMAPK1* is involved in shading stress, an RNA-Seq analysis was performed. The RNA used for the RNA-Seq was extracted from the leaves of the WT and *BnaMAPK1-*OE plants under normal light conditions and after 12 h of the shading treatment. Through pairwise comparisons, a total of 3,225, 3,000, 7,163, and 5,558 DEGs were, respectively, identified between the *BnaMAPK1-*OE and WT plants under normal light conditions (0h_OE vs. WT), *BnaMAPK1-*OE and WT under shading stress (12h_OE vs. WT), WT under normal light conditions and shading stress (WT_12h vs. 0 h), and *BnaMAPK1-*OE under normal light conditions and shading stress (OE_12h vs. 0 h), with a FDR < 0.05 and a |Log_2_FC| threshold > 1 (Figures 3A,B). In the 0h_OE vs. WT, 12h_OE vs. WT, WT_12h vs. 0 h, and OE_12h vs. 0 h groups, 1,904, 1,346, 3,213, and 2,302 DEGs were upregulated and 1,321, 1,654, 3,950, and 3,256 were downregulated in the shade treatment/overexpression line relative to the normal light condition/WT (Figure 3B). Moreover, using |Log_2_FC| > 3 as a more stringent cutoff, Figure 3C shows the 164 DEGs (accounting for 5.47% of all DEGs) in the 12h_OE vs. WT comparison, of which 85 were upregulated and 79 were downregulated.

### GO Enrichment Analysis of Photosynthesis-Related DEGs

**Figure 4:**
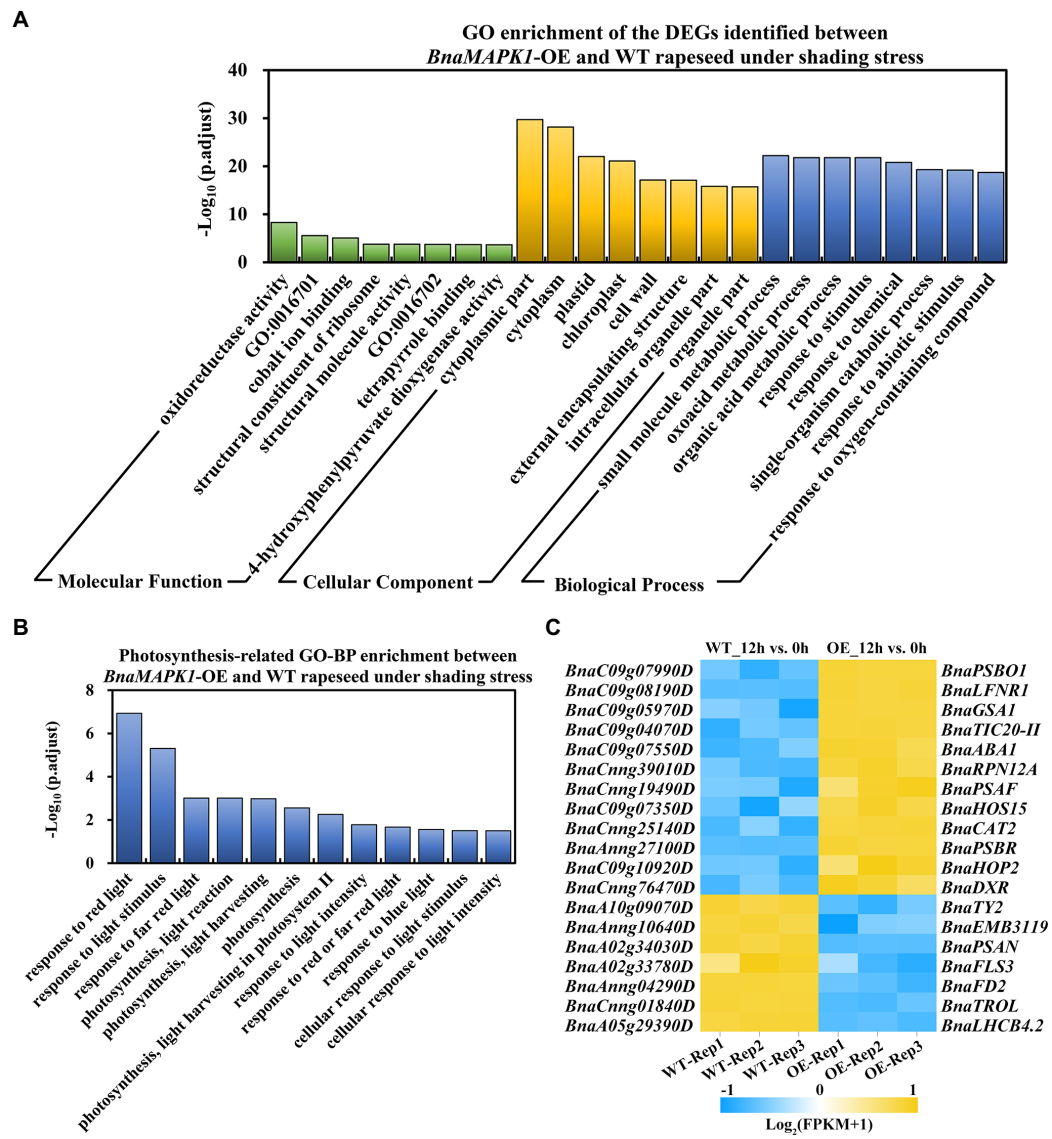
Significantly enriched GO pathways of DEGs in *BnaMAPK1*-OE and WT rapeseed under shading stress. **(A)** Distribution of top eight significantly enrich GO pathways in the 3,000 DEGs in 12h_OE vs. WT group (*p.adjust* < 0.05). GO categories included molecular function, cellular component, and biological process. GO:0016701, oxidoreductase activity, acting on single donors with incorporation of molecular oxygen; GO:0016702, oxidoreductase activity, acting on single donors with incorporation of molecular oxygen, incorporation of two atoms of oxygen. **(B)** GO analysis of DEGs involved in photosynthesis-related biological process in 12h_OE vs. WT group under shading stress (*p.adjust* < 0.05). **(C)** Heatmap depiction of the *BnaMAPK1* regulated photosynthesis-related DEGs in 12h_OE vs. WT group under shading stress. FPKM value of three replicates > 1, |Log_2_FC| > 3, and FDR < 0.05.

A GO enrichment analysis was performed on the 3,000 DEGs in the 12h_OE vs. WT group to annotate their functions into three main categories: molecular function (MF), cellular component (CC), and biological process (BP). Using a *p.adjust* < 0.05, a total of 570 GO pathways were assigned to the 3,000 DEGs that responded to shading stress, with 19, 83, and 468 in the MF, CC, and BP categories, respectively. Within GO-MF, “oxidoreductase activity (GO:0016491),” “oxidoreductase activity, acting on single donors with incorporation of molecular oxygen (GO:0016701),” and “cobalt ion binding (GO:0050897)” were the most dominant enriched terms. Within GO-CC, the significantly enriched terms were related to “cytoplasmic part (GO:0044444),” “cytoplasm (GO:0005737),” and “plastid (GO:0009536).” Within GO-BP, the majority of the enriched GO terms were involved in “small molecule metabolic process (GO:0044281),” “oxoacid metabolic process (GO:0043436),” and “organic acid metabolic process (GO:0006082).” The eight most significantly overrepresented GO terms in each category are shown in Figure 4A.

Given that the overexpression of *BnaMAPK1* improves photosynthesis, we focused on the GO-BP category differences between the *BnaMAPK1-*OE and WT plants that were implicated in the photosynthesis pathways, and identified 12 enriched GO pathways, including “response to light stimulus (GO:0009416),” “photosynthesis, light harvesting in PS II (GO:0009769),” and “response to light intensity (GO:0009642)” (Figure 4B; Supplementary Table S4). In these 12 photosynthesis-related GO-BP pathways, 276 DEGs were enriched in the 12h_OE vs. WT group, comprising 120 upregulated genes and 156 downregulated genes (Supplementary Table S5). According to the annotation, we found that the expression of *Rubisco activase* (*RCA*; *BnaA03g18710D*), *RAC-like 3* (*RAC*; *BnaA03g39270D*), *Early light-inducible protein* (*ELIP1*; *BnaCnng37300D*), and *Thioredoxin F2* (*TRXF2*; *BnaC02g06570D*) were significantly upregulated in *BnaMAPK1-*OE plants under shading stress, all of which encode the core subunits of PS I and PS II. We also observed that the key component of the light-harvesting antenna complex gene in PS II, *Light-harvesting complex photosystem II 4.2* (*LHCB4.2*; *BnaA05g29390D*), was significantly downregulated in the *BnaMAPK1-*OE plants compared with the WT under shaded conditions (Supplementary Table S5). Moreover, 19 DEGs were identified in this comparison using a |Log_2_FC| > 3 cutoff, of which 12 were upregulated and seven were downregulated (Figure 4C; Supplementary Table S5). These data indicated that *BnaMAPK1* may participate in photosynthesis by regulating the transcription of the photosystem complex subunits to improve the shading tolerance of rapeseed.

### KEGG Pathway Enrichment Analysis of the Photosynthesis-Related DEGs

**Figure 5:**
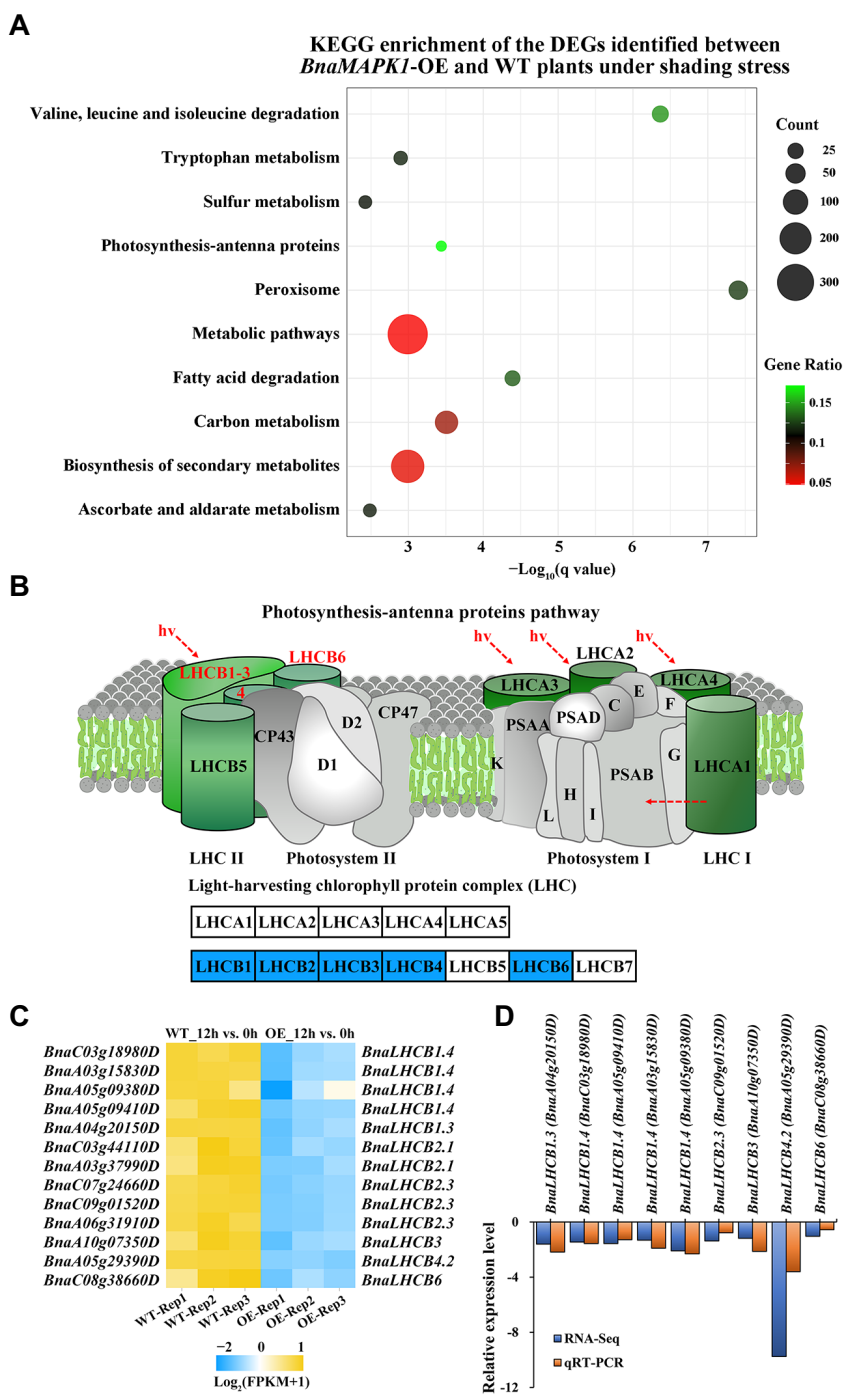
Photosynthesis-antenna proteins downregulated in *BnaMAPK1*-OE rapeseed relative to WT plants under shading stress in RNA-Seq. **(A)** Top 10 significantly enriched KEGG pathways in the 3,000 DEGs in 12h_OE vs. WT pairwise comparisons. The advanced bubble chart shows the enrichment of the DEGs in the signaling pathways. The *x*-axis label represents the enrichment significance (*q-value* < 0.05), and the *y*-axis label indicates the pathway names. The size of the circle represents the number of genes enriched in a particular pathway, and the color of bubble presents the enrichment factor (gene ratio = amount of DEGs enriched in the pathway/total number of genes in background gene set). **(B)** Downregulated *BnaLHCBs* encoding LHCB subunits in KEGG map of PS II-LHC II supercomplex in 12h_OE vs. WT pairwise comparisons. **(C)** Heatmap clustering of 13 *BnaLHCBs* transcript abundances associated with photosynthesis-antenna proteins pathway. Each DEG was screened with FPKM value of three replicates > 1, |Log_2_FC| > 1, and FDR < 0.05. **(D)** The relationship comparison between qRT-PCR and RNA-Seq fold change results.

For an exploration of the complex biological functions of the DEGs, the significantly enriched pathways were identified using KEGG analyses. The DEGs involved in these pathways are listed in Supplementary Table S3. The 3,000 DEGs from the 12h_OE vs. WT comparison were subjected to this KEGG characterization of their biological behaviors. In total, 18 KEGG pathways were obtained using a *q-value* < 0.05 (Supplementary Table S6). The top 10 KEGG pathways with the highest representation of DEGs are shown in Figure 5A, including “peroxisome (ko04146),” “valine, leucine and isoleucine degradation (ko00280),” “carbon metabolism (ko01200),” and “photosynthesis-antenna proteins (ko00196).” These data further confirmed the regulatory role of *BnaMAPK1* in the response to shading stress.

Based on the *BnaMAPK1-*mediated regulation of shading stress, we focused our analysis on 13 DEGs related to the “photosynthesis-antenna proteins” KEGG pathway, which includes genes related to PS II, such as *LHCB*. Based on their annotation, we found no obvious difference in the expression levels of genes encoding the PS I–LHC I supercomplex in the *BnaMAPK1-*OE plants compared with the WT under shading stress, while these 13 DEGs encoding LHCB subunits that function in the PS II–LHC II supercomplex were all significantly downregulated, especially *LHCB2.1* (*BnaC03g44110D*, *BnaA03g37990D*), *LHCB2.3* (*BnaC07g24660D*, *BnaC09g01520D*, *BnaA06g31910D*), *LHCB3* (*BnaA10g07350D*), *LHCB4.2* (*BnaA05g29390D*), and *LHCB6* (*BnaC08g38660D*) (Figures 5B,C). These data indicated that the expression of genes encoding LHCB subunits of the PS II–LHC II supercomplex was regulated by *BnaMAPK1* during shading stress.

### qRT-PCR Validation of the DEGs in the “Photosynthesis-Antenna Proteins” Pathway

To verify the authenticity of the expression levels obtained from the RNA-Seq data, the expression of nine selected *LHCB* genes were subjected to qRT-PCR analysis. *LHCB1.3* (*BnaA04g20150*), *LHCB1.4* (*BnaC03g18980*, *BnaA05g09410*, *BnaA03g15830*, *BnaA05g09380*), *LHCB2.3* (*BnaC09g01520*), *LHCB3* (*BnaA10g07350*), *LHCB4.2* (*BnaA05g29390*), and *LHCB6* (*BnaC08g38660*) were selected for validation, and *BnACT7* (*BnaC02g00690D*) was used as the internal control. The RNA templates for the qRT-PCR were obtained from 3 to 4-week-old mixed samples (leaf, stem, and root) of WT and *BnaMAPK1-*OE plants subjected to 0 or 12 h of the shading treatment. As shown in Figure 5D and Supplementary Figure S2, the changes in transcript expression revealed using RT-PCR were identical to those acquired using RNA-Seq. Collectively, the above expression results suggest that the RNA-Seq data were credible and that *BnaMAPK1* responds to shading stress by negatively regulating the *LHCB* genes in the PS II–LHC II supercomplex.

### Verification of the Physical Interaction of BnaLHC3 With BnaMAPK1 Using Y2H and Split-LUC System

**Figure 6:**
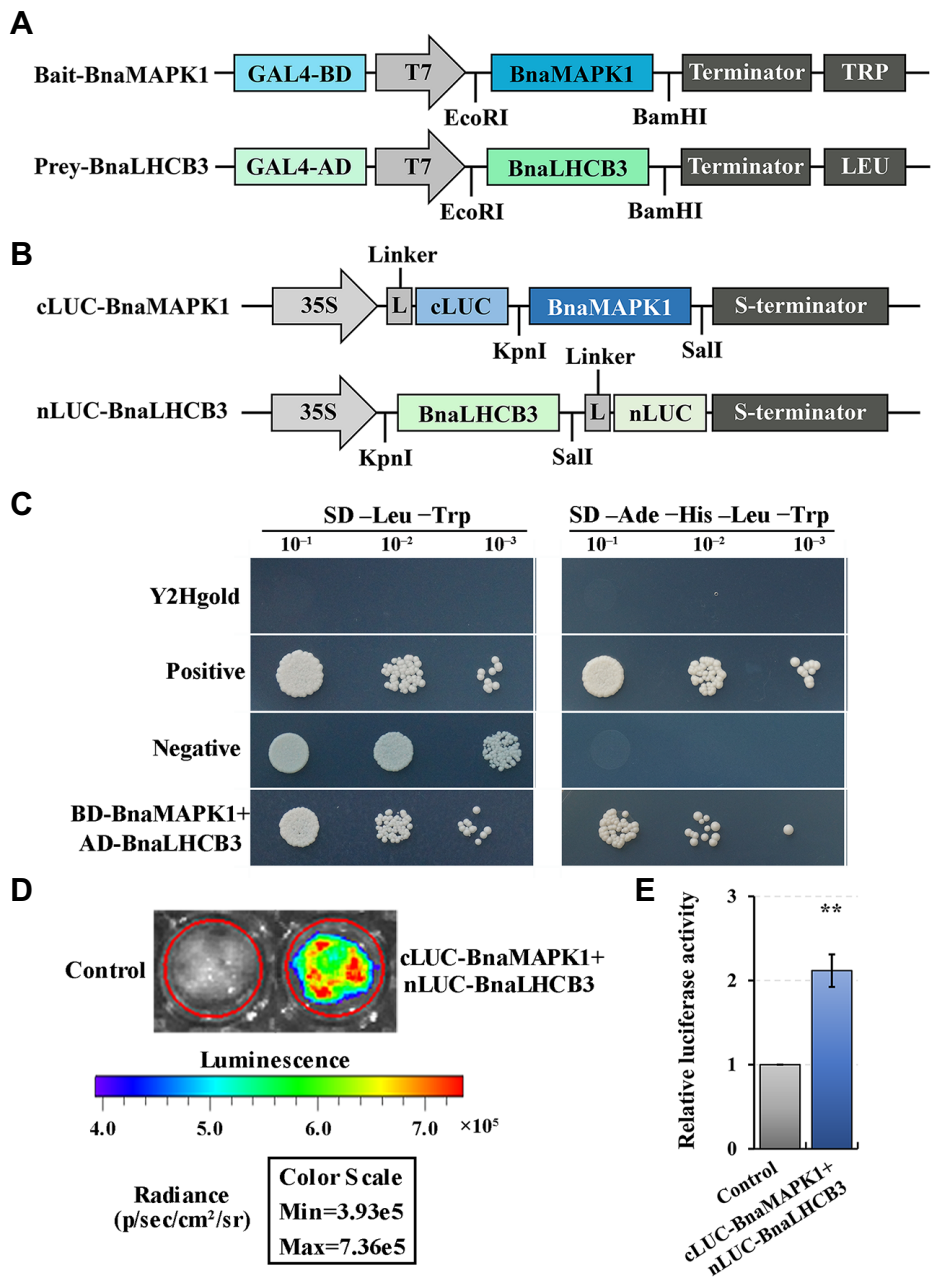
Verifying the interaction relationship of BnaMAPK1 with BnaLHCB3. **(A)** Schematic diagram of the expression vectors used in Y2H assay. **(B)** Schematic diagram of the expression vectors used in Split-LUC assay. **(C)** BnaMAPK1 interacted with BnaLHCB3 in Y2H assay. Y2Hgold cells served as blank controls, Y2Hgold cells expressing pGBKT7-53 and pGADT7-T served as positive controls, and Y2Hgold cells expressing pGBKT7-Lam and pGADT7-T served as negative controls. **(D)** BnaMAPK1 interacted with BnaLHCB3 in Split-LUC assay. Empty pCAMBIA1300-nLUC and pCAMBIA1300-cLUC were used as controls for normalization. **(E)** Relative luciferase activities assays for determining whether BnaMAPK1 could interact with BnaLHCB3 in tobacco leaves. All the experiments were repeated three times with similar results. Double asterisk above bars indicate significant difference at *p-value* < 0.01.

Our previous study revealed that a subunit of the PS II–LHC II supercomplex, BnaLHCB3 (BnaA10g07350D), is a candidate interaction partner of BnaMAPK1, as determined using Y2H library screening assays (Wang et al., 2020). To further explore whether BnaLHCB3 was regulated by BnaMAPK1, the full-length coding sequences of *BnaLHCB3* and *BnaMAPK1* were inserted into the pGADT7 and pGBKT7 vectors, respectively (Figure 6A). The prey and bait constructs were cotransformed into yeast to explore the point-to-point protein interactions. The Y2Hgold strain expressing *BnaLHCB3* and *BnaMAPK1* grew well on the SD–Ade-His-Leu-Trp medium (Figure 6C), indicating that BnaLHCB3 could interact with BnaMAPK1 in yeast cells.

To further elucidate the physical interaction of BnaLHCB3 and BnaMAPK1, tobacco-based firefly luciferase complementation assays were performed. *BnaLHCB3* was inserted into the N-terminal of LUC, while *BnaMAPK1* was linked to the C-terminal of LUC (Figure 6B). The coexpression of the nLUC-BnaLHCB3 and cLUC-BnaMAPK1 constructs showed a strong ability to rescue the luciferase enzyme activity (*p-value* < 0.01) (Figures 6D,E). Taken together, these results suggested that BnaLHCB3 and BnaMAPK1 interact *in vivo*, which might affect the stability of the PS II–LHC II supercomplex to further regulate shading tolerance in rapeseed.

## Discussion

Shade is widespread in nature, affecting many plants throughout their lifecycle. Plants respond on cellular and molecular levels to adapt to limited light. In our study, the AT1-motif, Box 4, G-box, GAG-motif, Sp1, TCT-motif, and As-2-box elements were identified in the promoter of *BnaMAPK1* (Supplementary Table S2), all of which are involved in the light-mediated transcriptional regulation of light-regulated genes in potato (*Solanum tuberosum*), maize (*Zea mays*), and *Arabidopsis thaliana* (Shariatipour and Heidari, 2020). Notably, a G-box in a promoter can bind tightly to phosphorylated proteins to negatively regulate the transcriptional expression of the *LHC II* genes to further respond to light in a variety of plants. This suggested that *BnaMAPK1* may play an important role in regulating photosynthesis in response to changing light levels.

### *BnaMAPK1* Regulates Light Capture Efficiency of the Photosynthetic Apparatus in Xanthophyll Cycle and Photosynthetic Pigment System

Previous studies show that the photosynthetic capability of shade-tolerant rapeseed is increased to extend the vegetative phase under a light-restricted environment (Brunel-Muguet et al., 2013), as well as optimized light capture and utilization through increased photosynthetic structures, primary reactions, and carbon assimilation in plants (Niinemets, 2010). Since the shading-induced expression pattern of *BnaMAPK1* was consistent with the *cis*-acting elements analysis of its promoter, we examined the photosynthetic structure of WT and *BnaMAPK1*-OE rapeseed under shade stress. In plants, Pn is the most important indicator for evaluating photosynthetic ability, especially under an artificial environment when it is provided with a constant light quality and intensity (Shengxin et al., 2016). Our data showed that the gas exchange values (Pn, Gs, Ci, and Tr) of both WT and *BnaMAPK1-*OE rapeseed decreased in the shading treatment, with the values in *BnaMAPK1-*OE higher than those in the WT (Figures 1A–D). Previously, however, silencing *Nicotiana attenuata NaMAPK4* (the homolog of *Arabidopsis AtMAPK4*) greatly enhances stomatal conductance, transpiration, and the photosynthetic rate in an ABA-dependent manner (Hettenhausen et al., 2012). Under shading stress, the decrease in Gs and Ci caused the decrease of Tr, which in turn caused the decrease of Pn, suggesting that *BnaMAPK1* plays a positive role in regulating the photosynthetic rate, which might be inconsistent with the mechanism by which *AtMAPK4* functions. In terms of energy dissipation, the trends of qP and Fv/Fm were similar to the gas exchange values, while the F0 and qN values of the WT and *BnaMAPK1-*OE plants increased after the shading treatment, with the WT displaying the higher values (Figures 2A–D). Photochemical damage is reflected in either an increase in F0 or decrease in the ratio of Fv/Fm under both shade stress and high-light conditions (Qiyuan and Ibrahim, 2016), implying that shading stress may limit the activity of the light reaction center and increase the energy dissipation in rapeseed. Our photosynthetic parameters demonstrated that *BnaMAPK1*-OE rapeseed maintained low heat dissipation to improve light capture efficiency to further enhance its photosynthetic rate under shading.

Xanthophylls play essential roles in both light absorption and photoprotection, acting as accessory pigments and structural elements of the antennae to stabilize the LHC II complex (Ballottari et al., 2012). In higher plants, the xanthophyll cycle is closely related to photosynthesis and photoinhibition under a shaded environment. This cycle mainly involves thermal energy dissipation, for which zeaxanthin epoxidase (ZEP) and violaxanthin de-epoxidase (VDE) are key catalyzing enzymes (Hieber et al., 2000). To gain a better understanding of how *BnaMAPK1* enhances photosynthetic capability under shading stress, GO pathway and related DEG analyses were performed in RNA-Seq. Our data showed that the expression of *Zeaxanthin epoxidase 1* (*ABA1/ZEP*; *BnaC09g07550D*) and *violaxanthin de-epoxidase-related protein* (*VDE*; *BnaCnng13890D*) were upregulated in transgenic rapeseed after the shading treatment (Supplementary Table S5), which was consistent with its photosynthetic traits. These data suggested that *BnaMAPK1* functions in the xanthophyll cycle of the photosynthetic apparatus to regulate energy dissipation and improve the light sensitivity of PS II.

Light affects the biosynthesis of the photosynthetic pigments, which in turn affects photosynthesis itself and plays an important role in plant growth and development. One such pigment, chlorophyll, absorbs and transmits light energy for photosynthesis (Sims and Gamon, 2002; Gao et al., 2020). In the present study, the relative chlorophyll contents of both WT and *BnaMAPK1-*OE were found to decrease under the shading treatment, but a long-term (14, 21, and 28-d) shading treatment resulted in significantly higher relative chlorophyll contents in *BnaMAPK1-*OE rapeseed compared with the WT (Figure 1E). Notably, three *Chlorophyllase 1* (*CLH1*; *BnaA02g18330D*, *BnaA06g13830D*, and *BnaC05g15260D*) genes, which are involved in chlorophyll degradation in PS II (Tian et al., 2021), were differentially expressed in the two genotypes, and were shown to be downregulated in the transgenic plants under shading stress. Here, the expression level of *BnaELIP1* (*BnaCnng37300D*), which belongs to the LHC-like protein family and is a target gene for controlling chlorophyll accumulation to enhance tolerance to high light and shading stresses (Heddad and Adamska, 2002), was upregulated in *BnaMAPK1-*OE rapeseed under the shading treatment (Supplementary Table S5). In *Arabidopsis* and *Chlamydomonas reinhardtii*, ELIP1 associates with LHCB antennae to function in the xanthophyll cycle, carbon assimilation, and chlorophyll accumulation (Stress et al., 2006; Lee et al., 2020). These findings indicated that the overexpression of *BnaMAPK1* reduces the damage caused to the photosynthetic pigments by shading stress, maintaining photosynthetic capability.

### *BnaMAPK1* Regulates Carbon Assimilation in Photosynthetic Enzyme System and Calvin Cycle in PS II

In addition to the photoreaction and pigment systems, the enzyme system is also an important component of photosynthesis. Rubisco is the major enzyme involved in carbon assimilation and the limiting factor of photosynthetic efficiency and productivity (Mitra and Baldwin, 2008). Lower levels of Rubisco, ATP synthase, and PS II activities, as well as less electron transport and CO_2_ consumption, are observed in shade-grown barley (*Hordeum vulgare*) leaves (Zivcak et al., 2014). By contrast, our photosynthetic measurements showed that the Rubisco activity in *BnaMAPK1-*OE rapeseed was significantly higher than the WT under both normal light and shade, which was positively correlated with the Pn values (Figure 2E). These findings are consistent with those of Liang et al., who report that shade-tolerant rapeseed has a higher photosynthetic net rate and chlorophyll content than the WT, as well as more stable Rubisco activity under shading stress (Liang and Li, 2004). Our results demonstrated that the photosynthetic enzyme system was less affected by shading in the *BnaMAPK1*-OE plants compared with WT rapeseed.

The Calvin cycle is responsible for photosynthetic carbon assimilation in plants. Relevant studies on shady environments reveal that the abundances of Rubisco, glyceraldehyde-3-phosphate dehydrogenase (GAPDH), and F-type ATPase, which are involved in the Calvin cycle, electron transport, and carbon assimilation, respectively, are reduced, suppressing photosynthesis (Zivcak et al., 2014; Liu et al., 2020). Rubisco catalyzes the first step of photosynthetic carbon assimilation and is a rate-limiting enzyme for photosynthetic efficiency and productivity in the Calvin cycle, in which Rubisco activity is regulated by a second enzyme, RCA (Portis, 2003; Bracher et al., 2017). Compared with the WT under shading stress, our RNA-Seq analysis showed that the expression level of *BnaRCA* (*BnaA03g18710D*) was upregulated in the *BnaMAPK1*-OE lines, which was consistent with the finding that overexpressing *BnaMAPK1* improved the Rubisco activity of shaded rapeseed. Moreover, previous studies report that GAPDH is involved in carbon assimilation and limited the regeneration of ribulose-1,5-bisphosphate (RuBP) in the Calvin cycle (Suzuki et al., 2021). We also found that *Glyceraldehyde-3-phosphate dehydrogenase C1* (*BnaGAPC1*; *BnaA09g46260D* and *BnaC08g40330D*) and *BnaGAPC2* (*BnaA05g33200D*), encoding GAPDH, were all significantly downregulated in the *BnaMAPK1-*OE lines, indicating that *BnaMAPK1* may play key roles in the Calvin cycle by accelerating RuBP carboxylation and limiting carbon assimilation. We therefore propose that the overexpression of *BnaMAPK1* is beneficial to shade-stressed rapeseed because it improves the efficiency of light energy conversion and allows for decreased energy dissipation during the carbon assimilation in PS II in a shaded environment.

### BnaMAPK1 Interacts With BnaLHCB3 to Participate in Photosynthesis-Antenna Proteins Pathway

Enhancing the photosynthetic rate is critical for increasing crop yields to meet the rising demands for food (Zhu et al., 2010; Long et al., 2015). The GO pathway analysis of the *BnaMAPK1-*OE lines in this study showed a significant enrichment in not only genes associated with the light reaction and harvesting in photosynthesis, but also those involved in plastoquinone biosynthesis (Figure 4; Supplementary Table S4). Of the DEGs, *Phytoene desaturation 1* (*BnaPDS1*; *BnaA09g49870D*, *BnaA10g04310D*, *BnaC05g04530D*, and *BnaC08g44820D*) and *Ferredoxin 3* (*BnaFD3*; *BnaA07g13260D*, *BnaC04g16810D*) were downregulated in *BnaMAPK1*-OE rapeseed compared with the WT under shading stress. *AtPDS1* was previously reported to function in plastoquinone biosynthesis, where it affects the availability of the electron carrier plastoquinone by controlling p-hydroxyphenylpyruvate dioxygenase activity during the light response in *Arabidopsis* (Qin et al., 2007). In higher plants, FD3 is a 2Fe2S plant-type iron–sulfur protein and associates with other ferredoxins to respond to a limitation in PS I acceptors (Voss et al., 2011). In the photoreaction process, ferredoxins act as electron acceptors of the photoreaction terminal, mediating electron transfer between PS I and carbon assimilation, and regulating electron reflow in photosynthetic phosphorylation (Fan et al., 2019). Electrons can be recycled from reduced ferredoxin at PS I to plastoquinone at PS II in the light-dependent reactions of photosynthesis (Munekage et al., 2004); therefore, based on the qP values presented here, it seems probable that *BnaMAPK1* influences the Calvin cycle and the xanthophyll cycle to further regulate electron transport to adapt to the shade, but is not highly likely to directly respond to an acceptor limitation at PS I or PS II in rapeseed.

Further mining our transcriptomic data, we conducted a KEGG pathway enrichment analysis to identify the potential genes and pathways involved in photosynthesis in the DEGs. We identified the enrichment of an important KEGG pathway, the photosynthesis-associated antenna proteins pathway (Figure 5; Supplementary Table S6). In higher plants and green algae, the chlorophyll-binding subunits of PS I and PS II are internal antennae; LHCs act as peripheral antennae that enable a more efficient absorption of light energy and rapidly transfer energy to the reaction center to improve the photosynthetic rate (Papadatos et al., 2017). Zhang’s lab discovers that AtMAPK3/AtMAPK6 activation can rapidly downregulate the PS II–LHC II supercomplex to inhibit photosynthesis (Su et al., 2018); however, combining the RNA-Seq and RT-PCR data, we identified an interesting group of DEGs encoding photosynthesis-associated antenna proteins in PS II, the BnaLHCBs, which are essential for photoprotection in both high-light and low-light stresses (Ballottari et al., 2012). These genes were significantly downregulated in the *BnaMAPK1*-OE rapeseed, including *BnaLHCB1*, *BnaLHCB2*, *BnaLHCB3*, *BnaLHCB4*, and *BnaLHCB6* (Figure 5; Supplementary Figure S2). In *Arabidopsis*, six *lhcb* single mutants, *lhcb1–6*, are investigated to show that each AtLHCB plays a specific role in photosynthesis and ABA signaling (Xu et al., 2012). Each of the five *BnaLHCB*s showed similar shading-sensitive expression patterns in our study, suggesting that all *BnaLHCB*s were necessary for building the antenna complex and keeping it intact. It is tempting to speculate that *BnaMAPK1* plays an important role in regulating the stability of the core molecular complex of the PS II antenna machinery, and that these *BnaLHCB*s might be the interacting partners of *BnaMAPK1* in regulating photosynthesis to improve the shade tolerance of rapeseed.

A previous proteomics analysis reports that differentially expressed proteins were also mapped to the photosynthesis-associated antenna proteins pathway; GmLHCB1–6 (except GmLHCB3) are significantly differentially abundant in soybean plants subjected to shading stress (Fan et al., 2019). Shading stress also induces PS state transitions (state 1–state 2 transitions), which maximize the efficiency of light harvesting at low light intensities (Mikko et al., 2006). However, LHCB3 affects the excitation energy transfer, the macrostructure of PS II, and the state transitions in *Arabidopsis* (Adamiec et al., 2015), and confers continuous-light tolerance and enhances yields in tomato (*Solanum lycopersicum*) and oil palm (Velez-Ramirez et al., 2014; Neoh et al., 2019). We previously use Y2H library screening assays to show that BnaLHCB3 is a candidate interacting partner of BnaMAPK1 (Wang et al., 2020). In the present study, we used point-to-point Y2H and split-LUC results to validate this interaction relationship between BnaMAPK1 and BnaLHCB3 (Figure 6), suggesting the function of BnaMAPK1 in stabilizing the PS II–LHC II supercomplex under shading stress in rapeseed.

**Figure 7:**
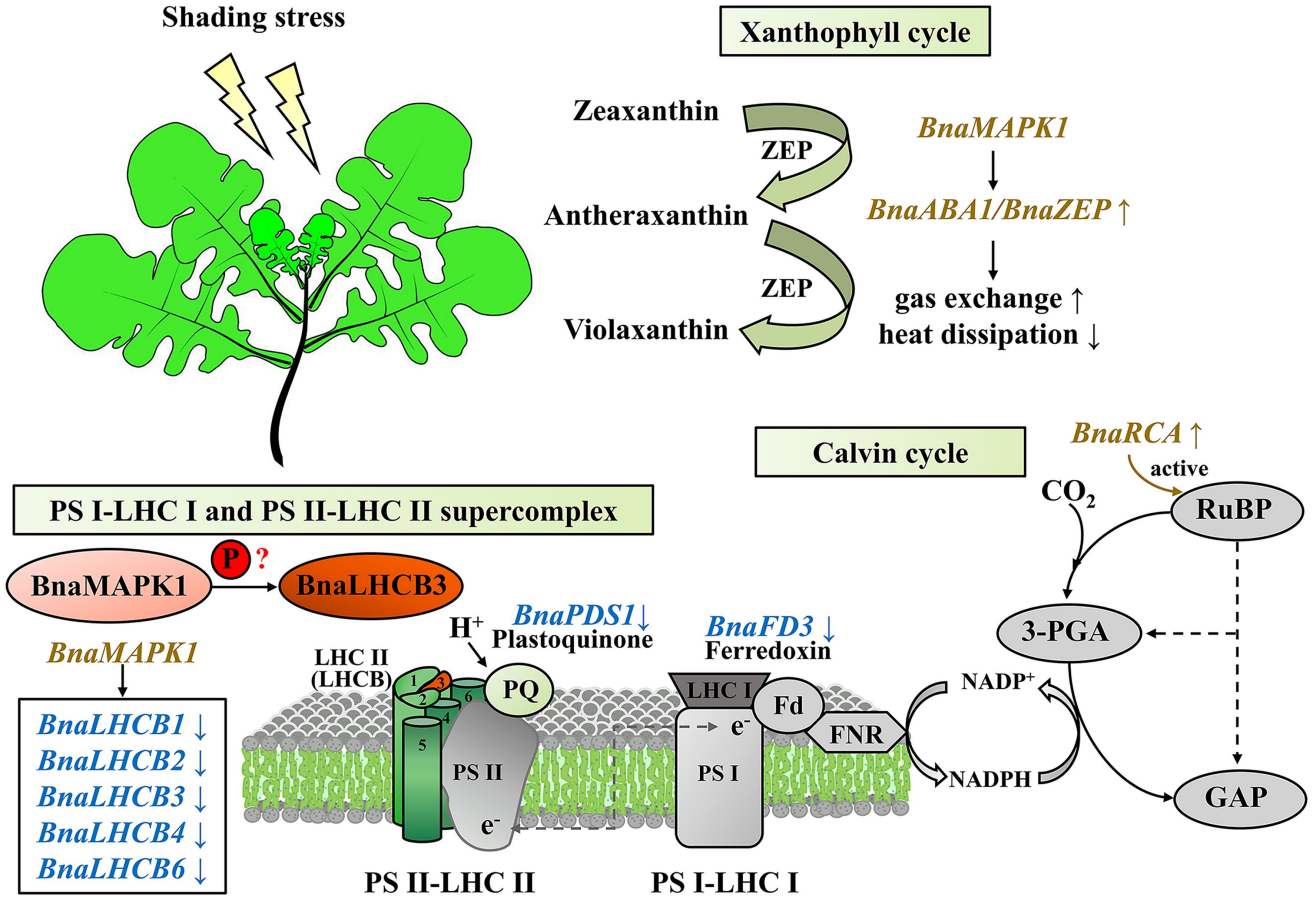
A proposed working model of *BnaMAPK1* functions in photosynthesis under shading stress in *Brassica napus*.

Taken together, our findings provide useful insights into the mechanisms underlying the rapeseed shading response through the exploration of *BnaMAPK1*, which is mainly involved in PS II-associated processes. This gene is particularly important in regulating the efficiency of the xanthophyll cycle and chlorophyll-mediated light capture and energy dissipation, controlling the Calvin cycle-mediated carbon assimilation, and stabilizing the PS II–LHC II supercomplex to improve photosynthesis capacity (Figure 7). This study provides important evidence for the molecular mechanisms underlying the shading response of the MAPK cascades, and is potentially relevant for use in molecular breeding strategies.

## Data Availability Statement

The datasets presented in this study can be found in online repositories. The names of the repository/repositories and accession number(s) can be found at: https://www.ncbi.nlm.nih.gov/bioproject/PRJNA680826/, PRJNA680826.

## Author Contributions

YL, LW, KL, and JL designed the research and supervised the project. ZW, ML, MY, and XZ performed the experiments. ZW, CQ, and HD analyzed the data. ZW and ML drafted the manuscript. ZW, LW, KL, and YL revised the manuscript. All authors contributed to the article and approved the submitted version.

## Funding

This work was funded by the National Natural Science Foundation of China (31872876 and 32101663), the China Postdoctoral Science Foundation (2021 M692683), the Chongqing Postdoctoral Natural Science Foundation (cstc2021jcyj-bshX0234), the Chongqing Special Funding in Postdoctoral Scientific Research (2010010006157688), the 111 Project of China (B12006), and the Agriculture Research System of Ministry of Finance and Minister of Agriculture and Rural Affairs of China.

## Conflict of Interest

The authors declare that the research was conducted in the absence of any commercial or financial relationships that could be construed as a potential conflict of interest.

## Publisher’s Note

All claims expressed in this article are solely those of the authors and do not necessarily represent those of their affiliated organizations, or those of the publisher, the editors and the reviewers. Any product that may be evaluated in this article, or claim that may be made by its manufacturer, is not guaranteed or endorsed by the publisher.
