# Supplementary material for: Rapeseed (Brassica napus) Mitogen-Activated Protein Kinase 1 Enhances Shading Tolerance by Regulating the Photosynthesis Capability of Photosystem II
Source: Front Plant Sci. 2022 Jun 2;13:902989. doi: 10.3389/fpls.2022.902989 (PMC9201689; doi:10.3389/fpls.2022.902989)
Supplement: Supplementary file 1 [file Data_Sheet_1.docx]

Supplementary Material

# Supplementary Figures and Tables

## Supplementary Figures


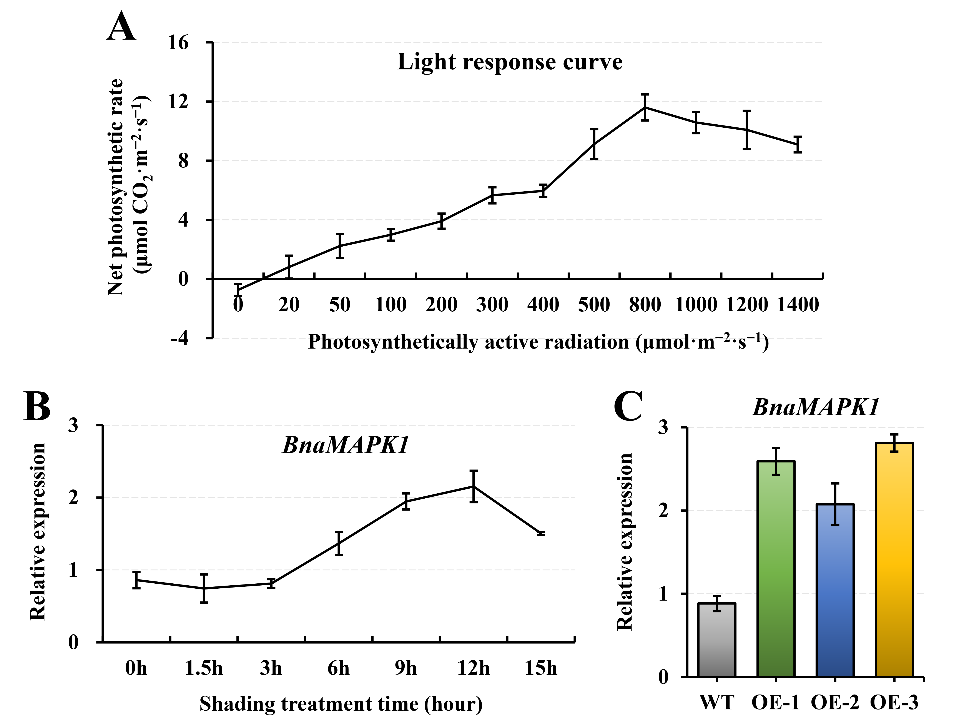


**Supplementary Figure 1.** ***BnaMAPK1* responses to shading stress in rapeseed.** A Light-photosynthesis curves of leaf in *Brassica napus* cultivar (Zhongyou821). Three seedlings were selected and three leaf were marked as fixed measured leaves, curves were measured ten times for each leaf. B Relative expression level of *BnaMAPK1* in Zhongyou821 rapeseed under shading treatment at 0, 1.5, 3, 6, 9, 12, 15 hours. C qRT-PCR analysis of the relative expression of *BnaMAPK1* in wild type and *BnaMAPK1*-overexpressing transgenic rapeseed. *BnaACT7* was used as in internal control for normalization. These data indicated means of three replicates ± SD.


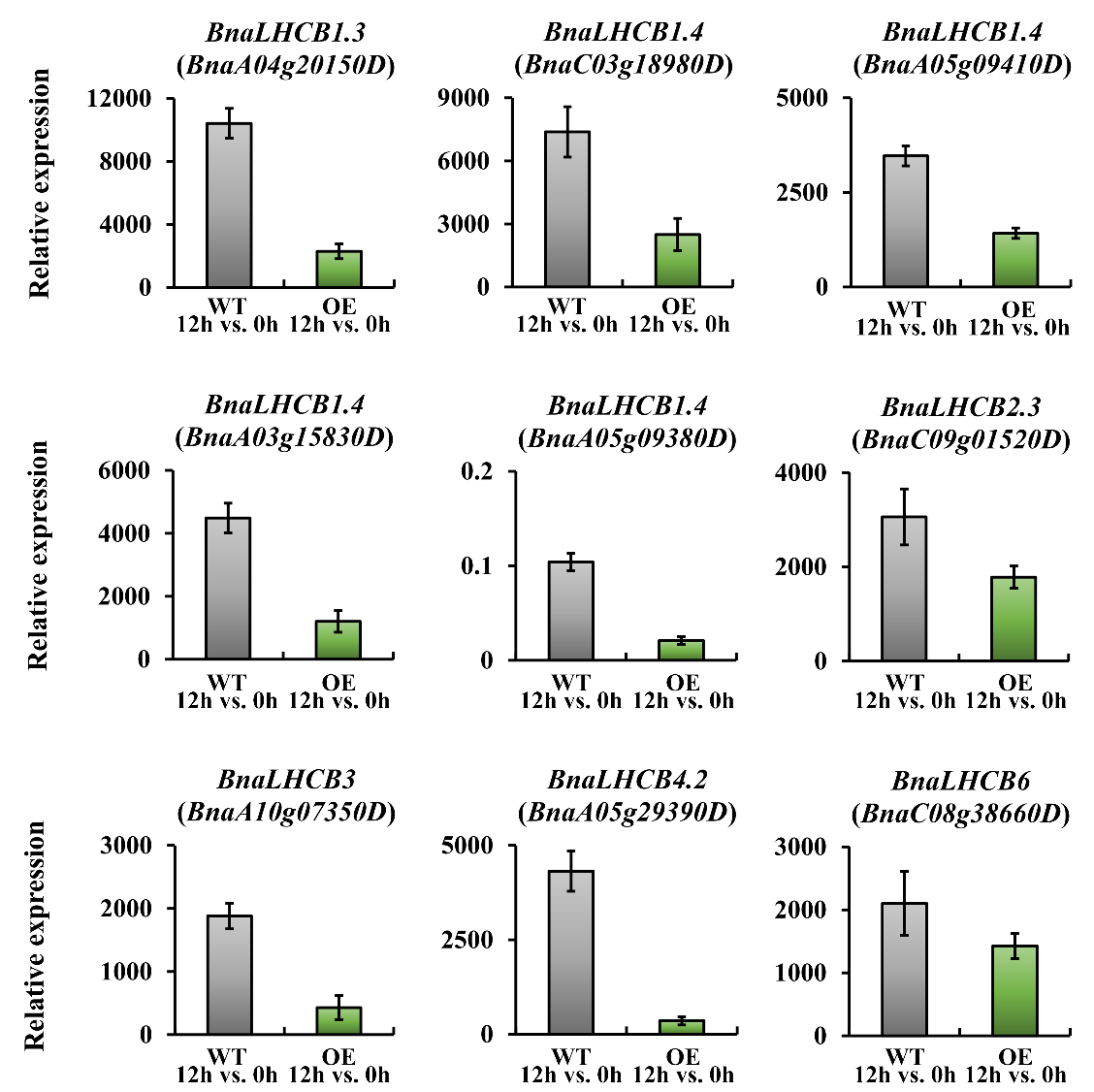


**Supplementary Figure 2.** **Validation of gene expression patterns in photosynthesis-antenna proteins pathway in wild type and *BnaMAPK1*-overexpressing transgenic rapeseed under shading stress.** Relative expression levels of *BnaLHCB1.3*, *BnaLHCB1.4*, *BnaLHCB2.3*, *BnaLHCB3*, *BnaLHCB4.2*, and *BnaLHCB6* under shading treatment compared with normal light condition via qRT-PCR. *BnaACT7* was used as in internal control for normalization. Vertical bars indicated means of three replicates ± SD.

## Supplementary Tables

**Table S1. Primers used for gene expression analysis and vector construction.**

| Primer name | Sequence (5’-3’) | Function |
| --- | --- | --- |
| BnaLHCB1.3_A04g20150-qF | TTCTCCTTCAGCATCAGAAGTCC | qRT-PCR primers of *BnaLHCB1.3* (*BnaA04g20150*) gene |
| BnaLHCB1.3_A04g20150-qR | TAGGTACTTGACTCTTTCGGAACC |  |
| BnaLHCB1.4_C03g18980-qF | TTGAAGGTTACAGAGTCGCCGGAGAA | qRT-PCR primers of *BnaLHCB1.4* (*BnaC03g18980*) gene |
| BnaLHCB1.4_C03g18980-qR | TGGGTCGGTAGCAAGACCCAACGGGTCA |  |
| BnaLHCB1.4_A05g09410-qF | AACCGCCCGTGTCACTATGCGC | qRT-PCR primers of *BnaLHCB1.4* (*BnaA05g09410*) gene |
| BnaLHCB1.4_A05g09410-qR | AACTTCTAGCTCACGGTTTCTTGCA |  |
| BnaLHCB1.4_A03g15830-qF | CGAGGTCTTTGGAACCGGGC | qRT-PCR primers of *BnaLHCB1.4* (*BnaA03g15830*) gene |
| BnaLHCB1.4_A03g15830-qR | TGAGGTAGCTCGGGGGCTCTCCA |  |
| BnaLHCB1.4_A05g09380-qF | AGCCATTTGGGCTACTCAGCC | qRT-PCR primers of *BnaLHCB1.4* (*BnaA05g09380*) gene |
| BnaLHCB1.4_A05g09380-qR | CCTTTAACTCTGCGAAGGCC |  |
| BnaLHCB2.3_C09g01520-qF | ACTTCAGCTATCCAACACTCCTCTTTTA | qRT-PCR primers of *BnaLHCB2.3* (*BnaC09g01520*) gene |
| BnaLHCB2.3_C09g01520-qR | TATGACGGTGTGTTCTCTGAGAATGGACCT |  |
| BnaLHCB3_A10g07350-qF | ATTCATGAGCTCAAGCAGTGTTTTGA | qRT-PCR primers of *BnaLHCB3* (*BnaA10g07350*) gene |
| BnaLHCB3_A10g07350-qR | ATCTCCCAAGTACACTATGGGAAATG |  |
| BnaLHCB4.2_A05g29390-qF | GGCTCCAGAGGTTCAGAGAGTGT | qRT-PCR primers of *BnaLHCB4.2* (*BnaA05g29390*) gene |
| BnaLHCB4.2_A05g29390-qR | CCATCCACTAGCTCTACCTTGCC |  |
| BnaLHCB6_C08g38660-qF | TTTGGGAACCGGCGTAGGCACTGG | qRT-PCR primers of *BnaLHCB6* (*BnaC08g38660*) gene |
| BnaLHCB6_C08g38660-qR | GTCTTTTCCCAAACCCAACGGGTCGA |  |
| BnaACT7-qF | TGGGTTTGCTGGTGACGAT | qRT-PCR primers of *BnaACT7* (*BnaC02g00690D*) gene |
| BnaACT7-qR | TGCCTAGGACGACCAACAATACT |  |
| BnaMAPK1-qF | GCTCAAGCTTCTACGCCATC | qRT-PCR primers of *BnaMAPK1* (*BnaA06g06010D*) gene |
| BnaMAPK1-qR | TCGAAGCAACTGGAACAAGA |  |
| BD-BnaMAPK1-F | cgGAATTCATGGCGACACCAGTTGAT | Y2H bait primers of *BnaMAPK1* gene |
| BD-BnaMAPK1-R | cgGGATCCTCAGAGCAGCTCAGATGGTGAA |  |
| Prey-BnaLHCB3-F | cgGAATTCATGGCTTCAACATTCATGAGCTCAAGC | Y2H prey primers of *BnaLHCB3* gene |
| Prey-BnaLHCB3-R | cgGGATCCTCAAGCACCAGGTACAAACTTTGTTGC |  |
| T7-F | TAATACGACTCACTATAGGG | Detection primers of Y2H pGBKT7 constructs |
| BD-R | TTTTCGTTTTAAAACCTAAGAGTC |  |
| AD-R | AGATGGTGCACGATGCACAG | Detection primers of Y2H pGADT7 constructs (with T7-F primer) |
| cLUC-BnaMAPK1-F | tacgcgtcccggggcGGTACCATGGCGACACCAGTTGAT | cLUC primers of *BnaMAPK1* gene |
| cLUC-BnaMAPK1-R | acgaaagctctgcagGTCGACTCAGAGCAGCTCAGATGGTGAA |  |
| nLUC-BnaLHCB3-F | cacggggacgagctcGGTACCATGGCTTCAACATTCATGAGCT | nLUC primers of *BnaLHCB3* gene |
| nLUC-BnaLHCB3-R | cgcgtacgagatctgGTCGACAGCACCAGGTACAAACTTTGTT |  |
| cLUC-F | CCGGAAAACTCGACGCAAGAAA | Detection primers of pCAMBIA1300-cLUC constructs |
| cLUC-R | CGAACGTTGTCGAAACCGATGATAC |  |
| nLUC-F | CACGGGGACGAGCTCGGTACC | Detection primers of pCAMBIA1300-nLUC constructs |
| nLUC-R | GGAACCAGGGCGTATCTCTTCA |  |

**Table S2. Identified main *cis*-acting regulatory elements and associated function in the promoter of *BnaMAPK1*.**

| Motifs | Organism | Sequence | Function |
| --- | --- | --- | --- |
| AAGAA-motif | *Avena sativa* | gGTAAAGAAA | // |
| AC-II | *Phaseolus vulgaris* | (C/T)T(T/C)(C/T)(A/C)(A/C)C(A/C)A(A/C)C(C/A)(C/A)C | homologous to AC-II element from the PAL2 gene from Phaseolus; element conferring enhanced xylem expression and repressed phloem expression in transgenic tobacco; enhancement of petal expression. |
| ARE | *Zea mays* | TGGTTT | cis-acting regulatory element essential for the anaerobic induction |
| AT1-motif | *Solanum tuberosum* | ATTAATTTTACA | part of a light responsive module |
| ATGCAAAT motif | *Oryza sativa* | ATACAAAT | cis-acting regulatory element associated to the TGAGTCA motif |
| Box 4 | *Petroselinum crispum* | ATTAAT | part of a conserved DNA module involved in light responsiveness |
| Box I | *Pisum sativum* | TTTCAAA | light responsive element |
| Box III | *Pisum sativum* | atCATTTTCACt | protein binding site |
| Box-W1 | *Petroselinum crispum* | TTGACC | fungal elicitor responsive element |
| CAAT-box | *Hordeum vulgare* | CAAT | common cis-acting element in promoter and enhancer regions |
| CGTCA-motif | *Hordeum vulgare* | CGTCA | cis-acting regulatory element involved in the MeJA-responsiveness |
| CTAG-motif | *Avena sativa* | ACTAGCAGAA | // |
| G-box | *Zea mays* | CACGTC | cis-acting regulatory element involved in light responsiveness |
| GAG-motif | *Arabidopsis thaliana* | AGAGAGT | part of a light responsive element |
| GT1-motif | *Arabidopsis thaliana* | GGTTAA | light responsive element |
| MBS | *Arabidopsis thaliana* | CAACTG | MYB binding site involved in drought-inducibility |
| Skn-1_motif | *Oryza sativa* | GTCAT | cis-acting regulatory element required for endosperm expression |
| Sp1 | *Zea mays* | CC(G/A)CCC | light responsive element |
| TATA-box | *Brassica napus* | ATTATA | core promoter element around -30 of transcription start |
| TC-rich repeats | *Nicotiana tabacum* | ATTCTCTAAC | cis-acting element involved in defense and stress responsiveness |
| TCT-motif | *Arabidopsis thaliana* | TCTTAC | part of a light responsive element |
| TGACG-motif | *Hordeum vulgare* | TGACG | cis-acting regulatory element involved in the MeJA-responsiveness |
| Unnamed__1 | *Zea mays* | CGTGG | // |
| Unnamed__3 | *Zea mays* | CGTGG | // |
| Unnamed__4 | *Petroselinum hortense* | CTCC | // |
| Unnamed__8 | *Glycine max cv. Provar* | CATTTTTGT | // |
| W box | *Arabidopsis thaliana* | TTGACC | elicitation; wounding and pathogen responsievness. Binds WRKY type transcription factors |
| As-2-box | *Nicotiana tabacum* | GATAatGATG | involved in shoot-specific expression and light responsiveness |
| Box E | *Petroselinum crispum* | ACCCATCAAG | cis-element for induction upon fungal elicitation |
| Circadian | *Lycopersicon esculentum* | CAANNNNATC | cis-acting regulatory element involved in circadian control |

**Table S3. Analysis of simulation and measured light response parameters in *Brassica napus*.**

|  | Light saturation point  (μmol·m^–2^·s^–1^) | Light compensation point  (μmol·m^–2^·s^–1^) | Dark respiration rate  (μmol·m^–2^·s^–1^) | Maximun net photosynthetic rate  (μmol·m^–2^·s^–1^) |
| --- | --- | --- | --- | --- |
| Ye Zi-Piao model | 886.19 | 9.65 | 0.82 | 6.52 |
| Measured data | 800 | 9.82 | 1.03 | 6.47 |

**Table S4. Enriched photosynthesis-related GO-BP pathways in *BnaMAPK1*-overexpressing and wild type plants under shading treatment.**

| GO ID | Description | p value | p.adjust | Out  (2768) | All  (79444) |
| --- | --- | --- | --- | --- | --- |
| GO:0010114 | response to red light | 2.89E-09 | 1.17E-07 | 42 | 430 |
| GO:0009416 | response to light stimulus | 2.16E-07 | 4.95E-06 | 227 | 4649 |
| GO:0010218 | response to far red light | 7.87E-05 | 0.00098 | 30 | 398 |
| GO:0019684 | photosynthesis, light reaction | 7.91E-05 | 0.00098 | 65 | 1133 |
| GO:0009765 | photosynthesis, light harvesting | 8.58E-05 | 0.00105 | 15 | 136 |
| GO:0015979 | photosynthesis | 0.00026 | 0.0028 | 76 | 1437 |
| GO:0009769 | photosynthesis, light harvesting in photosystem II | 0.00058 | 0.00557 | 4 | 12 |
| GO:0009642 | response to light intensity | 0.00207 | 0.0167 | 57 | 1096 |
| GO:0009639 | response to red or far red light | 0.00289 | 0.02148 | 78 | 1619 |
| GO:0009637 | response to blue light | 0.00394 | 0.02762 | 29 | 486 |
| GO:0071482 | cellular response to light stimulus | 0.00465 | 0.03142 | 20 | 300 |
| GO:0071484 | cellular response to light intensity | 0.0047 | 0.03158 | 5 | 32 |

**Table S5. List of photosynthesis-related DEGs identified between *BnaMAPK1*-overexpressing and wild type plants under shading condition.**

| Gene ID | Gene Name | WT-Rep1  FPKM | WT-Rep2  FPKM | WT-Rep3  FPKM | OE-Rep1  FPKM | OE-Rep2  FPKM | OE-Rep3  FPKM | FDR | Log_2_FC | Regulated |
| --- | --- | --- | --- | --- | --- | --- | --- | --- | --- | --- |
| BnaC09g07990D | PSBO1 | 1.495 | 0.175 | 1.208 | 8.432 | 8.261 | 8.302 | 0 | 8.162 | up |
| BnaC09g08190D | ATLFNR1 | 4.684 | 4.693 | 4.627 | 9.836 | 9.694 | 9.828 | 4E-266 | 5.102 | up |
| BnaC09g05970D | GSA1 | 1.173 | 0.992 | 0.049 | 4.541 | 4.480 | 4.495 | 6E-133 | 4.794 | up |
| BnaC09g04070D | - | 0.593 | 1.312 | 1.107 | 4.972 | 4.977 | 4.881 | 4E-99 | 4.770 | up |
| BnaC09g07550D | ABA1 | 0.927 | 1.002 | 1.230 | 2.742 | 2.733 | 2.586 | 2.2E-55 | 4.032 | up |
| BnaCnng39010D | RPN12a | 1.156 | 0.802 | 0.758 | 4.094 | 4.240 | 3.949 | 1.8E-68 | 3.872 | up |
| BnaCnng19490D | PSAF | 2.802 | 2.780 | 1.959 | 5.367 | 6.035 | 6.172 | 3.1E-12 | 3.543 | up |
| BnaC09g07350D | HOS15 | 2.383 | 2.106 | 2.528 | 3.495 | 3.616 | 3.507 | 1.2E-63 | 3.337 | up |
| BnaCnng25140D | CAT2 | 1.687 | 2.195 | 1.521 | 4.693 | 4.742 | 4.790 | 1.8E-94 | 3.269 | up |
| BnaAnng27100D | PSBR | 7.508 | 7.465 | 7.501 | 10.717 | 10.597 | 10.622 | 1E-120 | 3.144 | up |
| BnaC09g10920D | - | 1.246 | 1.256 | 0.965 | 2.501 | 2.926 | 2.805 | 1.9E-26 | 3.116 | up |
| BnaCnng76470D | DXR | 1.074 | 1.412 | 1.093 | 3.838 | 3.693 | 3.384 | 3.1E-19 | 3.082 | up |
| BnaC02g07030D | ENH1 | 2.939 | 2.896 | 2.852 | 5.600 | 5.436 | 5.432 | 1.1E-61 | 2.854 | up |
| BnaC04g53290D | AGL20 | 1.602 | 1.348 | 1.509 | 3.741 | 3.785 | 3.956 | 3E-35 | 2.750 | up |
| BnaA05g02620D | NUDX23 | 1.506 | 1.993 | 1.417 | 3.667 | 3.564 | 3.748 | 2.8E-41 | 2.682 | up |
| BnaC09g34100D | - | 4.914 | 4.926 | 4.941 | 7.508 | 7.276 | 7.403 | 2.2E-61 | 2.477 | up |
| BnaA10g18240D | - | 1.523 | 1.383 | 2.107 | 3.610 | 3.627 | 4.126 | 2E-09 | 2.470 | up |
| BnaCnng53550D | - | 0.303 | 1.086 | 0.000 | 2.249 | 1.818 | 1.662 | 6.7E-07 | 2.463 | up |
| BnaC02g08620D | APL1 | 1.062 | 1.513 | 0.608 | 2.424 | 2.281 | 3.375 | 6.2E-18 | 2.388 | up |
| BnaC09g50930D | DXR | 2.029 | 2.320 | 2.037 | 4.329 | 4.208 | 4.199 | 4.1E-45 | 2.370 | up |
| BnaC09g06120D | UVR8 | 2.406 | 2.796 | 2.894 | 4.919 | 4.943 | 4.807 | 1.2E-54 | 2.319 | up |
| BnaC09g12080D | CAT2 | 4.082 | 4.003 | 4.020 | 5.805 | 5.654 | 5.695 | 1.7E-59 | 2.292 | up |
| BnaC09g06600D | CESA6 | 3.374 | 3.123 | 3.319 | 5.288 | 5.441 | 5.466 | 5.8E-58 | 2.205 | up |
| BnaA03g02950D | PAS2 | 1.892 | 2.505 | 2.226 | 4.261 | 4.197 | 4.120 | 1.1E-32 | 2.192 | up |
| BnaC09g09900D | FVE | 1.314 | 1.322 | 1.731 | 2.606 | 2.750 | 2.828 | 2.4E-19 | 2.111 | up |
| BnaA01g04710D | IAA29 | 1.442 | 1.213 | 1.145 | 2.966 | 3.372 | 2.740 | 1.1E-06 | 2.101 | up |
| BnaAnng24970D | EDA3 | 1.680 | 1.831 | 0.994 | 3.380 | 3.024 | 3.154 | 1.1E-10 | 2.040 | up |
| BnaA03g38100D | ACO3 | 3.019 | 3.262 | 3.115 | 5.007 | 5.136 | 5.029 | 2E-47 | 2.018 | up |
| BnaA03g02320D | - | 4.773 | 4.866 | 4.668 | 6.836 | 6.747 | 6.765 | 3E-43 | 2.016 | up |
| BnaC09g02870D | RPS1 | 5.295 | 5.390 | 5.349 | 7.415 | 7.335 | 7.392 | 4.2E-51 | 2.016 | up |
| BnaA04g26320D | AGL20 | 1.461 | 1.139 | 0.714 | 2.722 | 2.583 | 2.248 | 6E-11 | 1.943 | up |
| BnaA06g05150D | CSD1 | 2.862 | 3.718 | 3.740 | 5.203 | 5.192 | 5.171 | 7.4E-34 | 1.903 | up |
| BnaC04g52510D | MAX2 | 1.031 | 1.177 | 1.251 | 2.412 | 2.556 | 2.508 | 1E-17 | 1.874 | up |
| BnaC04g40290D | GLR2.8 | 0.869 | 1.002 | 0.626 | 2.225 | 1.606 | 2.078 | 0.00016 | 1.832 | up |
| BnaA03g55720D | FTRA2 | 4.026 | 4.573 | 4.317 | 6.234 | 6.059 | 6.102 | 6.7E-33 | 1.828 | up |
| BnaA03g08160D | - | 2.608 | 2.128 | 2.754 | 4.211 | 3.967 | 4.360 | 1.6E-12 | 1.823 | up |
| BnaA02g33940D | UVR8 | 2.286 | 1.949 | 2.131 | 3.577 | 3.739 | 3.391 | 2.5E-25 | 1.819 | up |
| BnaCnng78230D | LOX2 | 1.863 | 2.172 | 2.324 | 3.776 | 3.651 | 3.648 | 1.1E-18 | 1.781 | up |
| BnaA05g37050D | DUF239 | 1.515 | 1.244 | 0.931 | 2.571 | 2.512 | 2.549 | 3.3E-12 | 1.774 | up |
| BnaC02g15950D | LOX2 | 0.730 | 0.757 | 1.053 | 2.068 | 1.909 | 1.599 | 1.8E-06 | 1.735 | up |
| BnaA03g18710D | RCA | 9.252 | 9.031 | 9.004 | 10.934 | 10.526 | 10.921 | 1.4E-14 | 1.682 | up |
| BnaA01g01400D | FAH1 | 2.992 | 3.035 | 2.814 | 4.263 | 4.695 | 4.594 | 5.5E-15 | 1.679 | up |
| BnaA03g51960D | IAA29 | 1.086 | 1.160 | 0.894 | 2.065 | 2.516 | 2.115 | 1.1E-06 | 1.676 | up |
| BnaC02g42580D | FLS3 | 1.988 | 2.419 | 2.248 | 3.801 | 3.739 | 3.582 | 1.1E-16 | 1.673 | up |
| BnaC04g48570D | PAR1 | 1.775 | 2.116 | 1.914 | 3.308 | 3.253 | 3.479 | 8.3E-07 | 1.640 | up |
| BnaC03g72320D | ELF4 | 2.370 | 2.834 | 2.808 | 3.991 | 4.352 | 4.262 | 1.5E-10 | 1.638 | up |
| BnaCnng71550D | EXPA1 | 4.883 | 4.908 | 4.789 | 6.595 | 6.393 | 6.468 | 7E-31 | 1.627 | up |
| BnaC05g43560D | OHP | 4.786 | 4.624 | 4.548 | 6.479 | 6.078 | 6.212 | 3.9E-15 | 1.616 | up |
| BnaCnng38640D | LOX2 | 1.545 | 1.591 | 1.320 | 2.594 | 2.747 | 2.668 | 5.3E-12 | 1.571 | up |
| BnaC06g05650D | - | 1.178 | 1.232 | 1.398 | 2.489 | 2.412 | 2.288 | 1.4E-09 | 1.555 | up |
| BnaC09g31000D | - | 0.864 | 1.014 | 1.004 | 2.082 | 1.808 | 1.901 | 0.0006 | 1.522 | up |
| BnaC04g21590D | CAT2 | 0.696 | 1.083 | 0.770 | 1.783 | 1.189 | 1.268 | 0.00041 | 1.520 | up |
| BnaA10g00700D | KCS1 | 2.080 | 2.285 | 2.152 | 3.319 | 3.595 | 3.530 | 6.5E-17 | 1.499 | up |
| BnaCnng37300D | ELIP1 | 0.976 | 1.297 | 0.973 | 2.169 | 2.117 | 1.777 | 0.00014 | 1.487 | up |
| BnaA01g07540D | DER1 | 2.182 | 2.246 | 2.167 | 3.545 | 3.514 | 3.436 | 1.3E-14 | 1.486 | up |
| BnaA06g05130D | MYB60 | 0.967 | 1.882 | 1.497 | 1.960 | 2.727 | 1.823 | 0.00346 | 1.476 | up |
| BnaC03g12530D | PHOT2 | 3.600 | 2.865 | 2.830 | 4.633 | 4.651 | 4.599 | 3.5E-26 | 1.460 | up |
| BnaA03g19900D | PAR1 | 1.691 | 1.562 | 1.264 | 2.688 | 2.835 | 2.401 | 0.0004 | 1.459 | up |
| BnaC09g23670D | AGL14 | 3.322 | 3.021 | 2.781 | 4.359 | 4.482 | 4.490 | 1.8E-15 | 1.453 | up |
| BnaA02g00920D | HY5 | 2.603 | 2.529 | 2.541 | 3.755 | 3.951 | 3.915 | 3E-09 | 1.417 | up |
| BnaC05g00780D | KCS1 | 2.834 | 2.851 | 3.132 | 4.248 | 4.268 | 4.258 | 4.3E-16 | 1.397 | up |
| BnaAnng29420D | NAP7 | 3.284 | 3.079 | 3.134 | 4.423 | 4.456 | 4.564 | 1.1E-15 | 1.387 | up |
| BnaC09g05250D | TOC1 | 3.402 | 3.004 | 3.212 | 4.614 | 4.587 | 4.263 | 4.9E-15 | 1.380 | up |
| BnaC05g29300D | IAA7 | 4.116 | 4.454 | 4.491 | 5.851 | 5.654 | 5.580 | 2.8E-21 | 1.378 | up |
| BnaA06g09110D | SAC52 | 3.026 | 3.167 | 2.936 | 4.371 | 4.366 | 4.292 | 6E-13 | 1.374 | up |
| BnaCnng02440D | EDA8 | 1.199 | 1.357 | 1.516 | 2.481 | 2.271 | 2.522 | 2.1E-06 | 1.371 | up |
| BnaA04g24760D | PIF4 | 1.267 | 0.836 | 0.883 | 1.833 | 1.926 | 1.470 | 4E-05 | 1.356 | up |
| BnaA09g20260D | ABC1 | 4.076 | 1.932 | 4.008 | 4.861 | 4.927 | 4.910 | 0.00027 | 1.349 | up |
| BnaA05g34990D | COP1 | 2.916 | 2.638 | 2.560 | 3.994 | 3.834 | 3.914 | 3.1E-17 | 1.307 | up |
| BnaA02g14340D | EXPA1 | 6.063 | 5.937 | 5.880 | 7.411 | 7.102 | 6.997 | 2.2E-11 | 1.296 | up |
| BnaA05g04540D | SEC61β | 2.547 | 3.263 | 3.361 | 4.424 | 4.291 | 4.242 | 3E-06 | 1.288 | up |
| BnaA01g04230D | LA1 | 1.056 | 0.776 | 1.196 | 1.683 | 1.897 | 1.767 | 0.00035 | 1.258 | up |
| BnaC05g02350D | KCS2 | 2.469 | 2.444 | 2.459 | 3.561 | 3.576 | 3.590 | 7.3E-13 | 1.247 | up |
| BnaA07g25310D | TSF | 2.334 | 2.275 | 1.886 | 3.353 | 3.184 | 3.238 | 8.8E-06 | 1.245 | up |
| BnaA05g34620D | RPL23AA | 4.021 | 3.890 | 4.196 | 5.317 | 5.123 | 5.293 | 2.5E-12 | 1.222 | up |
| BnaCnng32820D | CYP707A2 | 2.103 | 1.486 | 1.593 | 2.763 | 2.547 | 2.854 | 9.9E-05 | 1.217 | up |
| BnaA02g00400D | MIPS3 | 1.905 | 1.980 | 2.045 | 2.790 | 3.254 | 2.904 | 3.1E-10 | 1.212 | up |
| BnaC08g15350D | ELP | 5.407 | 4.969 | 4.815 | 6.641 | 6.026 | 6.150 | 0.0005 | 1.204 | up |
| BnaA06g33590D | CR88 | 4.471 | 4.516 | 4.403 | 5.765 | 5.636 | 5.581 | 9.8E-19 | 1.203 | up |
| BnaCnng24240D | - | 1.713 | 1.176 | 1.870 | 2.683 | 2.481 | 2.469 | 6E-06 | 1.196 | up |
| BnaC08g27940D | - | 1.058 | 1.227 | 1.179 | 1.940 | 1.967 | 1.975 | 0.00026 | 1.195 | up |
| BnaA10g02480D | KCS2 | 3.617 | 3.789 | 3.684 | 4.806 | 4.944 | 4.823 | 4.5E-17 | 1.191 | up |
| BnaC03g37680D | HSP70 | 1.010 | 1.173 | 1.188 | 1.730 | 1.904 | 2.049 | 1.1E-05 | 1.169 | up |
| BnaA05g34630D | PPL2 | 4.108 | 4.366 | 3.875 | 5.257 | 5.106 | 5.708 | 7.5E-05 | 1.168 | up |
| BnaA05g10140D | DAL1 | 2.164 | 2.223 | 2.209 | 3.334 | 3.230 | 3.057 | 4.3E-06 | 1.164 | up |
| BnaA06g37380D | MYB4 | 1.081 | 1.352 | 0.658 | 1.702 | 2.001 | 1.717 | 0.00245 | 1.162 | up |
| BnaC02g39790D | ACBP5 | 0.838 | 0.870 | 1.522 | 1.944 | 1.493 | 1.632 | 0.00015 | 1.146 | up |
| BnaC07g15280D | CAT3 | 7.372 | 7.446 | 7.560 | 8.709 | 8.853 | 8.828 | 2.9E-19 | 1.146 | up |
| BnaC08g36440D | AXR1 | 1.328 | 1.269 | 1.513 | 1.849 | 2.297 | 2.529 | 0.00253 | 1.144 | up |
| BnaC04g40260D | CYP707A2 | 1.959 | 1.571 | 1.565 | 2.806 | 2.464 | 2.513 | 4.2E-06 | 1.142 | up |
| BnaA03g34950D | ACT2 | 5.656 | 5.678 | 5.780 | 6.692 | 7.012 | 6.848 | 1E-17 | 1.137 | up |
| BnaC04g51700D | - | 2.131 | 1.970 | 1.827 | 2.951 | 3.023 | 2.787 | 9.7E-08 | 1.136 | up |
| BnaCnng49000D | - | 6.959 | 6.993 | 6.977 | 8.099 | 8.183 | 8.124 | 2.5E-18 | 1.131 | up |
| BnaA09g55900D | - | 5.190 | 5.260 | 5.305 | 6.417 | 6.266 | 6.480 | 7E-15 | 1.125 | up |
| BnaA06g29740D | PRMT4A | 1.382 | 1.357 | 1.124 | 2.259 | 2.087 | 2.151 | 1.5E-05 | 1.124 | up |
| BnaA06g01280D | NF-YA5 | 1.984 | 1.482 | 1.546 | 2.715 | 2.565 | 2.438 | 5.3E-05 | 1.114 | up |
| BnaA09g36350D | - | 1.526 | 1.781 | 1.545 | 2.385 | 2.702 | 2.346 | 0.00014 | 1.106 | up |
| BnaC08g35470D | - | 6.355 | 6.447 | 6.576 | 7.607 | 7.592 | 7.581 | 4.2E-16 | 1.106 | up |
| BnaC03g74470D | PGR5 | 5.545 | 5.493 | 5.589 | 6.812 | 6.494 | 6.655 | 8.8E-14 | 1.100 | up |
| BnaA03g41050D | - | 1.083 | 0.880 | 0.963 | 1.510 | 1.907 | 1.989 | 0.00264 | 1.097 | up |
| BnaA06g30590D | - | 2.312 | 2.869 | 2.361 | 3.248 | 3.700 | 3.507 | 2E-05 | 1.097 | up |
| BnaA09g28910D | UPS5 | 0.725 | 1.211 | 0.928 | 1.687 | 1.371 | 1.812 | 0.00661 | 1.090 | up |
| BnaA10g23080D | HEMC | 3.553 | 3.647 | 3.570 | 4.840 | 4.585 | 4.499 | 9.7E-12 | 1.090 | up |
| BnaC02g41600D | - | 1.029 | 2.234 | 2.343 | 2.707 | 3.050 | 2.908 | 0.00732 | 1.090 | up |
| BnaCnng69240D | CKA1 | 2.160 | 2.047 | 2.198 | 3.154 | 3.146 | 2.952 | 0.00048 | 1.084 | up |
| BnaC09g06130D | PP7 | 1.694 | 1.913 | 1.553 | 2.467 | 2.809 | 2.452 | 1.2E-05 | 1.074 | up |
| BnaA06g29970D | - | 4.436 | 4.027 | 4.038 | 5.322 | 5.197 | 5.182 | 9.1E-13 | 1.065 | up |
| BnaA06g27560D | KNAT3 | 4.378 | 4.478 | 4.408 | 5.372 | 5.808 | 5.500 | 1E-13 | 1.060 | up |
| BnaA07g28080D | EXPA1 | 3.648 | 3.817 | 3.561 | 4.792 | 4.621 | 4.738 | 1.5E-10 | 1.057 | up |
| BnaC07g09630D | - | 2.565 | 2.706 | 2.607 | 3.549 | 3.902 | 3.510 | 5.3E-09 | 1.042 | up |
| BnaC05g09420D | CLP2 | 3.754 | 4.039 | 4.024 | 5.101 | 4.923 | 4.877 | 5.1E-11 | 1.040 | up |
| BnaC05g44350D | - | 3.299 | 3.554 | 3.274 | 4.463 | 4.451 | 4.170 | 5E-09 | 1.037 | up |
| BnaC01g25860D | PRXCB | 2.320 | 2.552 | 2.420 | 3.264 | 3.410 | 3.367 | 8E-07 | 1.026 | up |
| BnaA06g14510D | CAT3 | 6.037 | 5.803 | 5.865 | 6.927 | 7.035 | 6.889 | 1.1E-14 | 1.026 | up |
| BnaA04g16900D | CYP707A2 | 1.530 | 1.243 | 1.260 | 2.477 | 2.030 | 1.849 | 0.00222 | 1.021 | up |
| BnaAnng32870D | - | 1.817 | 1.774 | 1.690 | 2.694 | 2.680 | 2.445 | 3.1E-06 | 1.014 | up |
| BnaA03g19740D | NUDX23 | 2.149 | 1.906 | 1.834 | 2.369 | 3.015 | 2.372 | 0.00201 | 1.014 | up |
| BnaCnng56050D | - | 1.882 | 1.698 | 1.514 | 2.540 | 2.367 | 2.586 | 3.1E-05 | 1.013 | up |
| BnaC05g47040D | - | 6.289 | 6.333 | 6.147 | 7.570 | 6.932 | 7.303 | 0.00047 | 1.010 | up |
| BnaA03g55930D | PPA6 | 3.356 | 3.424 | 3.275 | 4.221 | 4.307 | 4.390 | 1.1E-08 | 1.004 | up |
| BnaC09g50050D | - | 2.334 | 2.132 | 1.919 | 1.128 | 1.597 | 1.558 | 0.00565 | -1.000 | down |
| BnaA09g06690D | PSAN | 11.228 | 11.460 | 11.467 | 10.307 | 10.591 | 10.508 | 1.2E-15 | -1.006 | down |
| BnaA04g25070D | GLX2-1 | 4.781 | 4.955 | 4.936 | 3.878 | 4.116 | 3.977 | 5.3E-11 | -1.007 | down |
| BnaA09g48160D | ARR4 | 2.473 | 2.697 | 2.969 | 1.855 | 2.110 | 1.876 | 0.00034 | -1.008 | down |
| BnaA03g13280D | PABN1 | 4.393 | 4.513 | 4.458 | 3.516 | 3.715 | 3.376 | 3.8E-08 | -1.008 | down |
| BnaA06g32840D | TIP2 | 7.636 | 7.716 | 7.842 | 6.663 | 6.869 | 6.756 | 5.3E-14 | -1.009 | down |
| BnaC03g20200D | LOS2 | 4.337 | 4.799 | 4.797 | 3.754 | 3.846 | 3.602 | 2E-05 | -1.012 | down |
| BnaC03g03940D | ACT7 | 2.815 | 3.046 | 2.917 | 2.160 | 2.153 | 2.047 | 7.7E-06 | -1.014 | down |
| BnaC03g28340D | APX4 | 7.021 | 7.050 | 6.908 | 5.992 | 5.954 | 6.121 | 5.6E-14 | -1.014 | down |
| BnaA09g07350D | NPY3 | 3.475 | 3.140 | 3.025 | 2.404 | 2.420 | 2.366 | 8.9E-06 | -1.015 | down |
| BnaA03g37990D | LHCB2.1 | 6.581 | 6.890 | 6.870 | 5.765 | 5.772 | 5.892 | 4.7E-12 | -1.023 | down |
| BnaC08g11800D | RPN7 | 3.484 | 3.629 | 3.481 | 2.721 | 2.502 | 2.755 | 5E-08 | -1.027 | down |
| BnaC03g16960D | C4H | 1.489 | 1.760 | 1.958 | 1.079 | 1.080 | 1.211 | 0.00067 | -1.031 | down |
| BnaA09g53040D | CAT2 | 6.018 | 6.017 | 6.099 | 5.031 | 5.093 | 5.079 | 3.1E-14 | -1.033 | down |
| BnaC08g38660D | LHCB6 | 9.110 | 9.474 | 9.521 | 8.279 | 8.482 | 8.381 | 6.1E-08 | -1.034 | down |
| BnaAnng29760D | SPR1 | 4.748 | 4.599 | 4.421 | 3.390 | 3.838 | 3.658 | 1.3E-07 | -1.037 | down |
| BnaA09g47580D | CLPP6 | 2.667 | 2.650 | 2.898 | 2.146 | 1.816 | 1.899 | 3.4E-05 | -1.037 | down |
| BnaA09g03800D | - | 6.389 | 6.321 | 6.252 | 5.368 | 5.293 | 5.325 | 5.9E-14 | -1.045 | down |
| BnaC01g16110D | ACBP3 | 3.967 | 4.327 | 4.415 | 3.093 | 3.392 | 3.402 | 1.4E-06 | -1.047 | down |
| BnaA07g35300D | - | 4.742 | 4.843 | 5.073 | 3.909 | 4.000 | 3.864 | 5.1E-11 | -1.050 | down |
| BnaA06g26440D | CPSF100 | 3.398 | 3.296 | 3.065 | 2.375 | 2.469 | 2.313 | 4.8E-10 | -1.052 | down |
| BnaA03g13090D | LPA2 | 5.338 | 5.315 | 5.264 | 4.386 | 4.364 | 4.204 | 1.9E-10 | -1.056 | down |
| BnaA06g38220D | ELP | 7.471 | 7.086 | 7.079 | 6.466 | 6.155 | 5.966 | 3E-06 | -1.057 | down |
| BnaA09g04040D | CBP60G | 2.821 | 2.921 | 2.922 | 2.033 | 2.156 | 1.893 | 6.1E-08 | -1.061 | down |
| BnaC03g44110D | LHCB2.1 | 7.456 | 7.775 | 7.656 | 6.495 | 6.693 | 6.647 | 9.7E-14 | -1.063 | down |
| BnaA03g13310D | FSD2 | 2.944 | 2.738 | 2.527 | 1.809 | 1.656 | 1.554 | 4.5E-05 | -1.069 | down |
| BnaA05g07100D | TIM17-2 | 3.664 | 3.894 | 4.145 | 2.862 | 2.914 | 3.109 | 6.1E-06 | -1.080 | down |
| BnaC09g00690D | DFL2 | 3.062 | 2.748 | 2.949 | 2.133 | 1.925 | 2.044 | 8.9E-09 | -1.094 | down |
| BnaC08g11860D | RPN7 | 3.137 | 3.181 | 3.001 | 2.314 | 2.278 | 2.002 | 7.2E-11 | -1.096 | down |
| BnaA06g06660D | TIF3H1 | 3.139 | 3.385 | 3.108 | 2.093 | 2.207 | 2.278 | 4.8E-08 | -1.096 | down |
| BnaA03g31900D | ATKRS-1 | 3.159 | 3.197 | 3.224 | 2.320 | 2.321 | 2.239 | 1.2E-10 | -1.097 | down |
| BnaC02g01060D | - | 3.735 | 3.501 | 3.531 | 2.682 | 2.645 | 2.633 | 1.3E-11 | -1.097 | down |
| BnaA09g52370D | UVR8 | 5.423 | 5.489 | 5.372 | 4.381 | 4.471 | 4.453 | 5E-15 | -1.098 | down |
| BnaC09g28910D | - | 1.922 | 1.948 | 1.661 | 0.935 | 1.107 | 1.374 | 0.00221 | -1.110 | down |
| BnaA09g47800D | TIF3H1 | 4.975 | 5.219 | 5.241 | 4.038 | 4.304 | 3.970 | 8.9E-14 | -1.119 | down |
| BnaA06g31910D | LHCB2.3 | 5.341 | 5.456 | 5.322 | 4.278 | 4.300 | 4.382 | 7.5E-14 | -1.125 | down |
| BnaA09g50090D | STO | 3.582 | 3.522 | 3.613 | 2.808 | 2.585 | 2.262 | 2E-07 | -1.126 | down |
| BnaC07g47430D | FAH1 | 1.408 | 1.719 | 1.611 | 0.965 | 1.109 | 0.749 | 0.00067 | -1.132 | down |
| BnaA05g07240D | - | 8.616 | 8.590 | 8.530 | 7.564 | 7.484 | 7.402 | 5.7E-18 | -1.133 | down |
| BnaA06g28830D | - | 4.359 | 4.111 | 4.208 | 3.387 | 2.901 | 3.318 | 4.3E-12 | -1.138 | down |
| BnaC04g48190D | - | 2.712 | 2.695 | 3.056 | 2.346 | 1.569 | 1.906 | 9.9E-08 | -1.142 | down |
| BnaA04g21230D | PAL1 | 2.213 | 2.024 | 2.042 | 1.453 | 1.266 | 1.241 | 6.5E-05 | -1.154 | down |
| BnaA08g28860D | - | 2.253 | 2.451 | 2.583 | 1.595 | 1.875 | 1.239 | 0.00727 | -1.162 | down |
| BnaC02g17150D | - | 3.353 | 3.660 | 3.641 | 2.501 | 2.464 | 2.712 | 2.2E-06 | -1.162 | down |
| BnaA08g18790D | STZ | 2.979 | 2.565 | 2.329 | 1.795 | 1.801 | 1.687 | 0.00144 | -1.171 | down |
| BnaC08g29580D | PIL6 | 4.028 | 4.228 | 4.293 | 2.821 | 3.366 | 3.001 | 5.9E-14 | -1.173 | down |
| BnaA10g19390D | - | 6.271 | 6.434 | 6.295 | 5.167 | 5.010 | 5.440 | 4.1E-16 | -1.173 | down |
| BnaA09g06920D | HAM1 | 2.264 | 2.604 | 2.471 | 1.851 | 1.362 | 1.540 | 2.2E-06 | -1.180 | down |
| BnaA03g09250D | RHL41 | 2.778 | 2.912 | 3.100 | 1.976 | 2.054 | 1.972 | 0.00024 | -1.182 | down |
| BnaA10g07350D | LHCB3 | 7.641 | 7.958 | 7.873 | 6.528 | 6.742 | 6.787 | 4.5E-15 | -1.182 | down |
| BnaC04g28700D | - | 4.258 | 4.692 | 4.928 | 3.485 | 3.595 | 3.595 | 0.00018 | -1.194 | down |
| BnaCnng69530D | FSD2 | 1.958 | 1.608 | 1.620 | 0.775 | 0.951 | 1.264 | 0.0018 | -1.202 | down |
| BnaA09g03260D | HRB1 | 3.478 | 3.103 | 3.142 | 2.285 | 2.056 | 2.199 | 1.1E-06 | -1.202 | down |
| BnaCnng02320D | CCT | 2.164 | 1.686 | 1.706 | 1.057 | 1.140 | 0.969 | 1.9E-10 | -1.206 | down |
| BnaAnng11640D | CAT2 | 3.132 | 3.237 | 3.405 | 2.194 | 2.185 | 2.337 | 7.5E-12 | -1.206 | down |
| BnaA05g11810D | PSBP-1 | 2.918 | 3.292 | 3.047 | 2.090 | 1.832 | 2.305 | 2.8E-07 | -1.207 | down |
| BnaA03g38180D | XTH4 | 5.633 | 5.495 | 5.253 | 4.230 | 4.438 | 4.291 | 2E-13 | -1.221 | down |
| BnaA02g14510D | SAG24 | 5.047 | 5.580 | 5.773 | 3.302 | 5.084 | 4.452 | 0.00669 | -1.221 | down |
| BnaA09g05510D | COB | 5.215 | 5.237 | 5.230 | 3.946 | 4.030 | 4.050 | 1.6E-18 | -1.233 | down |
| BnaC02g31560D | CYP707A3 | 3.699 | 3.382 | 3.557 | 2.275 | 2.610 | 2.580 | 2.3E-12 | -1.248 | down |
| BnaA09g07840D | PSBO1 | 11.004 | 10.935 | 10.966 | 9.781 | 9.715 | 9.757 | 3.6E-23 | -1.252 | down |
| BnaC05g27870D | - | 2.343 | 2.107 | 2.374 | 1.423 | 1.623 | 1.110 | 0.00315 | -1.254 | down |
| BnaA05g06950D | SLT1 | 4.130 | 4.105 | 4.252 | 2.873 | 3.251 | 2.988 | 2.8E-15 | -1.255 | down |
| BnaA03g20220D | - | 2.724 | 2.729 | 2.839 | 1.879 | 1.908 | 1.559 | 7.3E-07 | -1.256 | down |
| BnaA06g33930D | PKS1 | 1.773 | 2.433 | 2.198 | 0.949 | 1.655 | 1.241 | 0.00842 | -1.259 | down |
| BnaA09g07020D | LIP1 | 2.931 | 3.301 | 3.319 | 2.259 | 2.466 | 2.285 | 1.3E-07 | -1.263 | down |
| BnaA03g12800D | CRR1 | 4.352 | 3.892 | 3.997 | 3.303 | 2.977 | 3.063 | 6.5E-10 | -1.274 | down |
| BnaA06g18100D | - | 3.773 | 4.363 | 4.523 | 2.850 | 3.441 | 2.975 | 0.00149 | -1.276 | down |
| BnaA05g06880D | EXP3 | 2.604 | 2.878 | 2.686 | 1.753 | 1.620 | 1.874 | 2.9E-06 | -1.279 | down |
| BnaC02g17140D | RBCS1A | 4.086 | 4.099 | 3.975 | 2.530 | 2.723 | 3.354 | 1.3E-09 | -1.282 | down |
| BnaA09g05810D | CNX1 | 5.046 | 4.970 | 4.958 | 3.809 | 3.802 | 3.798 | 3.6E-19 | -1.283 | down |
| BnaA09g07470D | HOS15 | 3.698 | 3.769 | 3.772 | 2.492 | 3.013 | 2.644 | 1.4E-16 | -1.288 | down |
| BnaA09g06460D | HDA6 | 3.612 | 3.658 | 3.717 | 2.534 | 2.501 | 2.632 | 1.3E-13 | -1.289 | down |
| BnaA06g39830D | SPR1 | 4.725 | 4.378 | 4.403 | 2.935 | 3.211 | 3.313 | 3.1E-13 | -1.301 | down |
| BnaA04g19490D | DAL1 | 3.201 | 3.402 | 2.935 | 2.193 | 2.051 | 2.117 | 3.8E-07 | -1.306 | down |
| BnaA09g12110D | CAT2 | 5.048 | 4.933 | 4.998 | 3.878 | 3.683 | 3.590 | 2.2E-20 | -1.308 | down |
| BnaA03g20470D | MPK6 | 3.525 | 3.432 | 3.485 | 2.739 | 2.482 | 2.750 | 7.2E-12 | -1.316 | down |
| BnaA03g15830D | LHB1B1 | 8.116 | 8.083 | 8.060 | 6.603 | 6.898 | 6.912 | 1.1E-23 | -1.317 | down |
| BnaC07g24660D | LHCB2.3 | 5.539 | 5.619 | 5.675 | 4.284 | 4.359 | 4.415 | 1.2E-19 | -1.335 | down |
| BnaAnng26580D | - | 3.339 | 3.497 | 3.774 | 2.451 | 2.559 | 2.208 | 3.4E-08 | -1.339 | down |
| BnaA03g13630D | - | 3.184 | 3.293 | 3.660 | 1.502 | 1.375 | 1.429 | 2E-08 | -1.352 | down |
| BnaA06g22530D | HDA6 | 1.582 | 1.747 | 1.734 | 0.903 | 1.017 | 0.817 | 6E-06 | -1.357 | down |
| BnaC02g00690D | ACT7 | 3.624 | 3.836 | 3.573 | 2.629 | 2.526 | 2.377 | 1.4E-12 | -1.363 | down |
| BnaC05g15970D | CAT3 | 2.150 | 2.062 | 2.111 | 1.036 | 1.240 | 1.329 | 1.6E-09 | -1.369 | down |
| BnaC09g01520D | LHCB2.3 | 8.400 | 8.483 | 8.493 | 7.072 | 7.109 | 7.200 | 5.7E-26 | -1.371 | down |
| BnaA04g25200D | - | 7.211 | 7.237 | 7.235 | 5.522 | 5.032 | 6.141 | 9.3E-24 | -1.377 | down |
| BnaA10g26930D | PRR7 | 3.153 | 3.095 | 3.007 | 2.265 | 2.049 | 1.826 | 5.4E-16 | -1.381 | down |
| BnaC08g16850D | HSP70b | 1.616 | 1.866 | 1.574 | 0.775 | 1.146 | 0.737 | 1.1E-06 | -1.401 | down |
| BnaA02g32550D | PRR5 | 2.989 | 2.489 | 2.868 | 1.676 | 1.688 | 1.662 | 1.5E-08 | -1.430 | down |
| BnaA09g52380D | PP7 | 3.086 | 3.320 | 3.381 | 2.041 | 2.016 | 1.963 | 7.9E-14 | -1.436 | down |
| BnaA10g25000D | DREB2A | 2.071 | 1.586 | 1.970 | 0.863 | 1.115 | 1.031 | 4.3E-05 | -1.438 | down |
| BnaC06g40210D | - | 4.120 | 4.439 | 4.413 | 2.618 | 3.347 | 3.040 | 2.8E-11 | -1.445 | down |
| BnaC01g13470D | SKS4 | 1.710 | 1.795 | 1.708 | 0.667 | 1.175 | 0.811 | 1.8E-06 | -1.449 | down |
| BnaA03g56740D | COR15A | 2.834 | 3.604 | 2.917 | 1.655 | 2.240 | 2.017 | 0.00664 | -1.453 | down |
| BnaC03g18980D | LHB1B1 | 9.966 | 9.853 | 9.977 | 8.299 | 8.575 | 8.652 | 2E-30 | -1.453 | down |
| BnaA03g09920D | TIL | 5.261 | 5.378 | 5.275 | 4.115 | 3.958 | 3.716 | 1E-18 | -1.460 | down |
| BnaC04g53590D | SLT1 | 4.486 | 4.793 | 4.868 | 3.248 | 3.415 | 3.398 | 1.4E-14 | -1.496 | down |
| BnaA06g29350D | RPS1 | 5.676 | 5.633 | 5.628 | 4.296 | 4.181 | 4.186 | 1.2E-26 | -1.507 | down |
| BnaA08g22890D | - | 1.530 | 2.273 | 2.067 | 0.849 | 1.098 | 1.095 | 0.00248 | -1.533 | down |
| BnaA04g24630D | - | 6.661 | 6.689 | 6.694 | 4.758 | 5.429 | 5.315 | 6.9E-31 | -1.546 | down |
| BnaA05g09410D | LHB1B1 | 7.862 | 8.143 | 8.177 | 6.432 | 6.594 | 6.612 | 7.1E-22 | -1.564 | down |
| BnaA05g22960D | HCF107 | 4.637 | 4.441 | 4.303 | 3.067 | 2.969 | 3.167 | 3E-24 | -1.570 | down |
| BnaA04g24160D | CYP83A1 | 5.010 | 4.430 | 4.227 | 3.333 | 3.093 | 3.020 | 0.00039 | -1.583 | down |
| BnaC08g41760D | SEC22 | 2.964 | 3.276 | 3.237 | 2.430 | 1.702 | 2.077 | 7.1E-14 | -1.596 | down |
| BnaA05g21210D | NAD | 6.802 | 6.517 | 6.467 | 5.221 | 4.946 | 5.172 | 6.4E-23 | -1.599 | down |
| BnaA04g20150D | CAB1 | 12.167 | 12.152 | 12.168 | 10.399 | 10.622 | 10.740 | 1.7E-38 | -1.603 | down |
| BnaA05g21300D | RAB7B | 4.629 | 4.802 | 4.776 | 3.525 | 3.531 | 3.468 | 4.4E-23 | -1.635 | down |
| BnaC08g45150D | - | 1.774 | 1.790 | 1.774 | 0.655 | 0.823 | 1.023 | 0.00247 | -1.645 | down |
| BnaA03g36950D | IAA7 | 4.144 | 4.250 | 4.304 | 2.696 | 2.798 | 2.738 | 3.7E-18 | -1.674 | down |
| BnaC01g02790D | ACO1 | 4.933 | 5.088 | 5.028 | 3.435 | 3.520 | 3.353 | 1.3E-34 | -1.700 | down |
| BnaA09g06230D | DXR | 3.995 | 3.864 | 3.838 | 2.449 | 2.201 | 2.557 | 2.4E-26 | -1.720 | down |
| BnaAnng40540D | CNX1 | 4.326 | 4.198 | 4.169 | 3.475 | 2.216 | 1.826 | 2.9E-07 | -1.742 | down |
| BnaA05g05290D | - | 4.963 | 4.776 | 4.645 | 3.007 | 3.094 | 3.169 | 2.1E-25 | -1.776 | down |
| BnaC08g04750D | SBP1 | 2.745 | 2.887 | 2.775 | 1.030 | 1.579 | 1.266 | 6.9E-12 | -1.799 | down |
| BnaA05g05680D | - | 4.644 | 4.726 | 4.395 | 2.712 | 2.769 | 2.783 | 1.2E-23 | -1.810 | down |
| BnaC08g39130D | CPN10 | 5.631 | 5.902 | 5.770 | 4.195 | 4.004 | 3.860 | 4.9E-24 | -1.844 | down |
| BnaA01g14640D | - | 1.316 | 1.966 | 1.530 | 0.328 | 0.456 | 1.076 | 0.0037 | -1.862 | down |
| BnaC04g05080D | CKB4 | 2.825 | 2.622 | 2.982 | 1.186 | 1.175 | 1.246 | 6.1E-13 | -1.867 | down |
| BnaA05g02780D | EMB2184 | 3.710 | 3.808 | 3.690 | 2.241 | 2.008 | 2.132 | 2.3E-12 | -1.886 | down |
| BnaC01g03420D | CAT2 | 2.959 | 2.858 | 3.113 | 1.449 | 2.088 | 1.438 | 3.9E-12 | -1.907 | down |
| BnaAnng30740D | RPN12a | 4.135 | 4.102 | 4.305 | 2.465 | 2.590 | 2.415 | 8.9E-24 | -1.925 | down |
| BnaC05g03150D | - | 3.641 | 3.695 | 3.455 | 1.890 | 1.970 | 2.052 | 1.1E-13 | -1.954 | down |
| BnaA05g36800D | BCAT4 | 3.209 | 2.567 | 3.014 | 1.545 | 1.255 | 1.429 | 1.3E-07 | -1.971 | down |
| BnaA05g06760D | LCD1 | 4.418 | 4.369 | 4.370 | 2.709 | 2.508 | 2.618 | 3.4E-34 | -1.990 | down |
| BnaCnng57040D | CYP707A3 | 3.523 | 2.851 | 3.030 | 0.907 | 1.857 | 1.854 | 1.6E-05 | -1.994 | down |
| BnaA06g15140D | - | 5.329 | 5.778 | 5.905 | 3.511 | 4.083 | 3.639 | 1.9E-10 | -2.006 | down |
| BnaAnng34280D | CAO | 6.838 | 6.884 | 6.881 | 4.959 | 4.977 | 4.849 | 2.5E-48 | -2.008 | down |
| BnaA07g33680D | NIA1 | 3.590 | 3.662 | 3.510 | 2.193 | 1.783 | 1.732 | 5.6E-20 | -2.022 | down |
| BnaCnng62600D | XTH4 | 5.550 | 5.345 | 5.404 | 3.705 | 3.324 | 3.499 | 3.4E-41 | -2.048 | down |
| BnaA02g04780D | DIN10 | 5.538 | 5.520 | 5.648 | 3.323 | 4.003 | 3.499 | 1.8E-49 | -2.058 | down |
| BnaA05g09380D | LHB1B1 | 2.844 | 2.868 | 2.408 | 0.208 | 1.234 | 1.790 | 8.9E-07 | -2.094 | down |
| BnaC04g41120D | C4H | 2.022 | 2.772 | 2.316 | 0.942 | 1.042 | 0.956 | 5.1E-05 | -2.138 | down |
| BnaA05g22300D | - | 5.923 | 6.043 | 5.918 | 4.008 | 3.971 | 3.785 | 3.5E-45 | -2.154 | down |
| BnaA05g23690D | FAD5 | 6.376 | 6.231 | 6.207 | 4.228 | 4.151 | 3.990 | 6.4E-57 | -2.252 | down |
| BnaA04g17560D | C4H | 1.891 | 2.580 | 2.862 | 0.946 | 0.929 | 1.101 | 0.00047 | -2.261 | down |
| BnaC01g02910D | SEN1 | 5.168 | 5.138 | 5.581 | 3.024 | 3.223 | 3.253 | 5.5E-16 | -2.301 | down |
| BnaC05g33030D | BCAT4 | 3.524 | 2.979 | 3.229 | 1.451 | 1.332 | 1.610 | 5.2E-13 | -2.315 | down |
| BnaA07g33690D | NIA1 | 3.005 | 2.955 | 3.041 | 1.345 | 0.971 | 1.459 | 4.2E-40 | -2.334 | down |
| BnaA05g03290D | ETFQO | 3.003 | 3.296 | 3.364 | 1.294 | 1.513 | 1.452 | 5.5E-25 | -2.345 | down |
| BnaA01g34290D | PB | 2.000 | 2.076 | 2.552 | 0.578 | 1.271 | 0.268 | 1.2E-05 | -2.406 | down |
| BnaA03g02640D | ATAF2 | 4.237 | 4.383 | 4.436 | 2.101 | 2.329 | 2.061 | 2.6E-45 | -2.420 | down |
| BnaA03g18840D | ELF4 | 3.839 | 4.030 | 3.808 | 1.837 | 1.928 | 1.502 | 9.8E-18 | -2.553 | down |
| BnaC03g34940D | BCE2 | 4.575 | 5.345 | 5.261 | 2.609 | 2.700 | 2.774 | 7.4E-09 | -2.635 | down |
| BnaCnng72260D | ASN1 | 2.587 | 3.309 | 3.672 | 0.765 | 1.480 | 1.305 | 4.1E-06 | -2.637 | down |
| BnaA05g22450D | - | 4.729 | 5.141 | 5.322 | 2.487 | 2.784 | 2.584 | 4.4E-15 | -2.703 | down |
| BnaA02g34170D | CPuORF40 | 1.900 | 2.032 | 1.719 | 1.178 | 1.198 | 0.698 | 2.6E-22 | -2.728 | down |
| BnaA01g01780D | SEN1 | 8.143 | 8.910 | 8.953 | 5.666 | 6.126 | 6.117 | 7.9E-09 | -2.781 | down |
| BnaCnng01910D | GBF2 | 3.804 | 4.030 | 3.857 | 1.338 | 1.511 | 1.489 | 2.4E-69 | -2.982 | down |
| BnaA06g17510D | ASN1 | 3.222 | 4.024 | 4.074 | 1.328 | 1.399 | 1.502 | 2.2E-08 | -2.994 | down |
| BnaA10g09070D | TY2 | 4.668 | 4.502 | 4.627 | 1.971 | 1.701 | 2.169 | 1.9E-33 | -3.031 | down |
| BnaAnng10640D | - | 4.219 | 4.290 | 4.131 | 1.010 | 1.864 | 1.909 | 2.4E-51 | -3.063 | down |
| BnaA02g34030D | PSAN | 7.795 | 7.651 | 7.815 | 4.387 | 4.463 | 4.453 | 3E-116 | -3.257 | down |
| BnaA02g33780D | FLS3 | 2.620 | 3.348 | 3.210 | 1.243 | 0.582 | 0.413 | 1.3E-29 | -3.357 | down |
| BnaAnng04290D | FED A | 8.476 | 8.496 | 8.438 | 3.413 | 3.191 | 2.789 | 1E-246 | -5.513 | down |
| BnaCnng01840D | TROL | 8.228 | 8.141 | 8.157 | 2.711 | 2.597 | 3.026 | 8E-290 | -5.637 | down |
| BnaA05g29390D | LHCB4.2 | 10.674 | 10.905 | 10.983 | 1.669 | 1.903 | 1.421 | 4E-259 | -9.752 | down |

**Table S6. Enriched KEGG pathways of the DEGs in *BnaMAPK1*-overexpressing and wild type plants under shading treatment.**

| KEGG ID | KEGG A class | KEGG B class | Description | Out  (833) | All  (19835) | Q value |
| --- | --- | --- | --- | --- | --- | --- |
| ko04146 | Cellular Processes | Transport and catabolism | Peroxisome | 42 | 338 | 0.0000 |
| ko00280 | Metabolism | Amino acid metabolism | Valine, leucine and isoleucine degradation | 28 | 190 | 0.0000 |
| ko00071 | Metabolism | Lipid metabolism | Fatty acid degradation | 23 | 173 | 0.0000 |
| ko01200 | Metabolism | Global and overview maps | Carbon metabolism | 76 | 1093 | 0.0003 |
| ko00196 | Metabolism | Energy metabolism | Photosynthesis-antenna proteins | 13 | 76 | 0.0004 |
| ko01110 | Metabolism | Global and overview maps | Biosynthesis of secondary metabolites | 222 | 4183 | 0.0010 |
| ko01100 | Metabolism | Global and overview maps | Metabolic pathways | 370 | 7532 | 0.0010 |
| ko00380 | Metabolism | Amino acid metabolism | Tryptophan metabolism | 18 | 154 | 0.0013 |
| ko00053 | Metabolism | Carbohydrate metabolism | Ascorbate and aldarate metabolism | 16 | 139 | 0.0033 |
| ko00920 | Metabolism | Energy metabolism | Sulfur metabolism | 16 | 142 | 0.0038 |
| ko00620 | Metabolism | Carbohydrate metabolism | Pyruvate metabolism | 28 | 340 | 0.0062 |
| ko00072 | Metabolism | Lipid metabolism | Synthesis and degradation of ketone bodies | 5 | 18 | 0.0066 |
| ko00350 | Metabolism | Amino acid metabolism | Tyrosine metabolism | 16 | 153 | 0.0066 |
| ko00945 | Metabolism | Biosynthesis of other secondary metabolites | Stilbenoid, diarylheptanoid and gingerol biosynthesis | 7 | 37 | 0.0066 |
| ko00330 | Metabolism | Amino acid metabolism | Arginine and proline metabolism | 22 | 250 | 0.0072 |
| ko03010 | Genetic Information Processing | Translation | Ribosome | 82 | 1389 | 0.0081 |
| ko01210 | Metabolism | Global and overview maps | 2-Oxocarboxylic acid metabolism | 23 | 289 | 0.0185 |
| ko00640 | Metabolism | Carbohydrate metabolism | Propanoate metabolism | 12 | 117 | 0.0250 |
